# Clinical predictive models for recurrence and survival in treated laryngeal and hypopharyngeal cancer: a systematic review and meta-analysis

**DOI:** 10.3389/fonc.2024.1478385

**Published:** 2024-12-06

**Authors:** Ahmad K. Abou-Foul, Janine Dretzke, Esther Albon, Caroline Kristunas, David J. Moore, Andreas Karwath, Georgios Gkoutos, Hisham Mehanna, Paul Nankivell, the PETNECK2 Research Team

**Affiliations:** ^1^ Institute for Head and Neck Studies and Education, University of Birmingham, Birmingham, United Kingdom; ^2^ Department of Cancer and Genomic Sciences & Centre for Health Data Science, University of Birmingham, Birmingham, United Kingdom; ^3^ Department of Applied Health Research, University of Birmingham, Birmingham, United Kingdom

**Keywords:** outcome predictive model, laryngeal cancer, hypopharyngeal cancer, survival, recurrence, systematic review

## Abstract

**Background:**

The limitations of the traditional TNM system have spurred interest in multivariable models for personalized prognostication in laryngeal and hypopharyngeal cancers (LSCC/HPSCC). However, the performance of these models depends on the quality of data and modelling methodology, affecting their potential for clinical adoption. This systematic review and meta-analysis (SR-MA) evaluated clinical predictive models (CPMs) for recurrence and survival in treated LSCC/HPSCC. We assessed models’ characteristics and methodologies, as well as performance, risk of bias (RoB), and applicability.

**Methods:**

Literature searches were conducted in MEDLINE (OVID), Embase (OVID) and IEEE databases from January 2005 to November 2023. The search algorithm used comprehensive text word and index term combinations without language or publication type restrictions. Independent reviewers screened titles and abstracts using a predefined Population, Index, Comparator, Outcomes, Timing and Setting (PICOTS) framework. We included externally validated (EV) multivariable models, with at least one clinical predictor, that provided recurrence or survival predictions. The SR-MA followed PRISMA reporting guidelines, and PROBAST framework for RoB assessment. Model discrimination was assessed using C-index/AUC, and was presented for all models using forest plots. MA was only performed for models that were externally validated in two or more cohorts, using random-effects model. The main outcomes were model discrimination and calibration measures for survival (OS) and/or local recurrence (LR) prediction. All measures and assessments were preplanned prior to data collection.

**Results:**

The SR-MA identified 11 models, reported in 16 studies. Seven models for OS showed good discrimination on development, with only one excelling (C-index >0.9), and three had weak or poor discrimination. Inclusion of a radiomics score as a model parameter achieved relatively better performance. Most models had poor generalisability, demonstrated by worse discrimination performance on EV, but they still outperformed the TNM system. Only two models met the criteria for MA, with pooled EV AUCs 0.73 (95% CI 0.71-0.76) and 0.67 (95% CI 0.6-0.74). RoB was high for all models, particularly in the analysis domain.

**Conclusions:**

This review highlighted the shortcomings of currently available models, while emphasizing the need for rigorous independent evaluations. Despite the proliferation of models, most exhibited methodological limitations and bias. Currently, no models can confidently be recommended for routine clinical use.

**Systematic review registration:**

https://www.crd.york.ac.uk/prospero/display_record.php?ID=CRD42021248762, identifier CRD42021248762.

## Introduction

Laryngeal and hypopharyngeal squamous cell carcinomas (LSCC/HPSCC) are a complex subset of head and neck cancer (HNC) that have poor prognosis, and whose treatment poses a significant impact to patients’ health and quality of life. The complexity of balancing treatment efficacy with the preservation of critical functions like speech and swallowing represents a significant challenge for clinicians and patients (1).

Traditionally, the American Joint Committee on Cancer/International Union Against Cancer (AJCC/UICC) TNM classification system formed the cornerstone for risk-stratification in LSCC/HPSCC patients (2–4). However, this system only uses limited clinical features, and has inherent limitations such as the inability to factor in response to treatment to provide patient-level predictions (2, 5–7). To address these limitations, there is a growing interest in developing more sophisticated multi-variable clinical predictive models (CPMs), incorporating clinical features, molecular biomarkers, and radiomic signatures to augment the accuracy of prognostication. However, previous systematic reviews (SRs) have underscored the limitations in existing prognostic models for mixed HNCs, as well as their considerable risk for bias (8, 9). The efficacy of CPMs hinges inherently on the characteristics of included patients, the quality of the datasets employed in their development, and the rigor of modelling methodology. These factors may profoundly shape the models’ performance and applicability, and influence the implementation of such models in routine clinical practice (8).

The primary objective of this systematic review and meta-analysis (SR/MA) is to perform for the first time, a comprehensive evaluation of externally validated CPMs for survival and/or recurrence in adults with LSCC/HPSCC.

## Methods

The SR was reported according to the Preferred Reporting Items for Systematic Reviews and Meta-analyses (PRISMA) guidelines (10), and the recently published Transparent reporting of multivariable prediction models for individual prognosis or diagnosis - systematic reviews and meta-analyses (TRIPOD-SRMA) (11). A protocol was registered with PROSPERO (CRD42021248762).

### Information sources and search strategy

A comprehensive literature search of the MEDLINE, MEDLINE In Process (OVID), Embase (OVID) and the IEEE databases was conducted. We included articles published between January 2005 and November 2023, with no restrictions on language, or the age and sex of the target population. Initially, we imposed no restrictions on publication type, but excluded letters to editors and conference abstracts that lacked sufficient details on modelling techniques and performance assessment. The search algorithm used comprehensive text word and index term combinations relating to LSCC/HPSCC and prognostic models (**Supplementary Table 1A**). Terms for prognostic models were based on the search strategy proposed by Geersing et al. (12). Additionally, we searched reference lists of included studies, and additional studies were included if deemed eligible.

### Study selection process

Titles and abstracts were independently screened by at least two reviewers (AA-F, JD, EA, DM) using Rayyan software (www.rayyan.ai), and following eligibility criteria based on the PICOTS (13) framework (population, index, comparator, outcome, timing and setting), **Table 1**. Only externally validated multivariable models, that included at least one clinical variable in the final model, were included. Eligible models can either be individualized predictions models (IPMs), or risk stratification models (RSMs) that only classified patients into broader risk categories. Studies using multivariate analysis to identify predictors significantly associated with an outcome but not attempting to develop a model were excluded. Disagreements were resolved through consensus, or referral to a wider expert steering committee.

**Table 1:** Population, Index, Comparator, Outcomes, Timing and Setting (PICOTS) framework for the systematic review’s scope and eligibility criteria for inclusion.

POPULATION
Adults (≥18 years) with LSCC or/and HPSCC who have completed treatment with curative intent.
INDEX MODEL
All models that combined two or more predictors (prognostic factors) in a statistical model to provide individualized cancer survival and/or recurrence predictions, or categorize patients into risk groups according to risk of recurrence or/and death. *AND* Models’ predictors need to include clinical variables (e.g. age, sex, tumour staging parameters, smoking/alcohol consumption, etc.), with or without additional molecular biomarkers or radiomics variables. *AND* Developed models must be externally validated at least once in the same study (TRIPOD type 3 validation (36)) or in a separate publication (TRIPOD type 4 validation (36). External validation (EV) was defined as validation in a separate patient cohort from a different institution or registry. Models that were reported in development-only studies, but were externally validated in a separate studies were included. *Models with only molecular biomarkers or radiomic variables were excluded.* *Models that were only internally validated, or lacked external validation, were excluded.* *Models developed using national cancer registry data (e.g. SEER), and only validated on randomly selected cohort from the same registry were excluded (no true geographical EV).*
COMPARATOR
Benchmarking performance against the TNM system was desirable but not an eligibility criterion
OUTCOME
Any recurrence or survival related outcomes (e.g., Recurrence, local control (LC), overall survival (OS), etc.)
TIMING
Any prediction ‘time-zero’ was allowed (e.g., pre-treatment or post-treatment) Any prediction ‘horizon’ was allowed (e.g., 1-year OS, 5-year LC, etc.)
SETTING
No restriction on treatment setting or intended model use.

### Data items and collection process

Key data was collected using a pre-designed and piloted data extraction form (A-AF, JD, EA), based on the Critical Appraisal and Data Extraction for Systematic Reviews of Prediction Modelling Studies (CHARMS) checklist (14). Disagreements were resolved through consensus. We collected data on patient characteristics for each development and external validation (EV) cohort, in addition to details on study design, and final model variables. We also extracted data on model performance measures for discrimination (e.g., C-index and/or the area under the curve (AUC)), calibration (e.g., calibration plots), overall model fit and accuracy (e.g., Brier’s scores), and clinical utility if reported.

### Risk of bias and applicability assessment

The Prediction model Risk of Bias Assessment Tool (PROBAST) (13, 15) was used to assess risk of bias (RoB) and applicability concerns for each model. PROBAST assesses RoB across four domains (participants, predictors, outcomes, and analysis), and applicability concerns across three domains (participants, predictors, and outcomes). A domain-level RoB judgment of ‘high’, ‘unclear’ or ‘low’ concern was given to each model, and an overall judgment for each model was made (13).

### Synthesis methods

Models’ discrimination was assessed using reported C-index and/or AUC with these widely accepted thresholds: 0.5–0.59 (poor), 0.6–0.69 (weak), 0.7–0.79 (good), 0.8–0.89 (very good) and ≥0.9 (excellent) (16, 17). Quantitative pooling of performance measures from different models was deemed clinically meaningless and methodologically flawed due to differences in population, length of follow-up, and performance metrics. Discrimination metrics for all models were presented in forest plots without quantitative pooling. For models that were externally validated in more than two cohorts, meta-analysis (MA) for EV performance (discrimination and calibration) was planned for each model independently, as per the framework for MA of prediction models proposed by Debray et al. (18, 19). This framework recommends using a random effects model with restricted maximum likelihood estimation, and the Sidik-Jonkman Hartung-Knapp method for constructing the pooled confidence interval. We also calculated the 95% prediction intervals to estimate potential model performance in a new EV, and estimated the probability of good performance (AUC/C-index ≥ 0.7) when the model is applied in practice to a new unseen population (18, 20). We measured inter-study heterogeneity using the Cochran’s *Q* test and *I* ^2^ statistic, with significant heterogeneity defined as chi-square *p <*0·05 or *I* ^2^ >50% (21, 22).

We also assessed the change in discrimination ability from development to EV for each model, by calculating the delta AUC (dAUC), or delta C-index (dCI), representing the absolute difference between derivation and EV values. Additionally, we computed the percentage of change in discriminative ability on EV (dAUC% or dCI%), relative to the reference AUC/C-index value of 0.50 (random chance) (23). For example, if an AUC drops from 0.80 in derivation to 0.65 in validation, the dAUC will be -0.15, and dAUC% will be 50% decrease in discriminative ability (23). We used R statistical software (v4.3.1), and “metamisc’ package (v0.4.0) (19).

## Results

**Figure 1:**
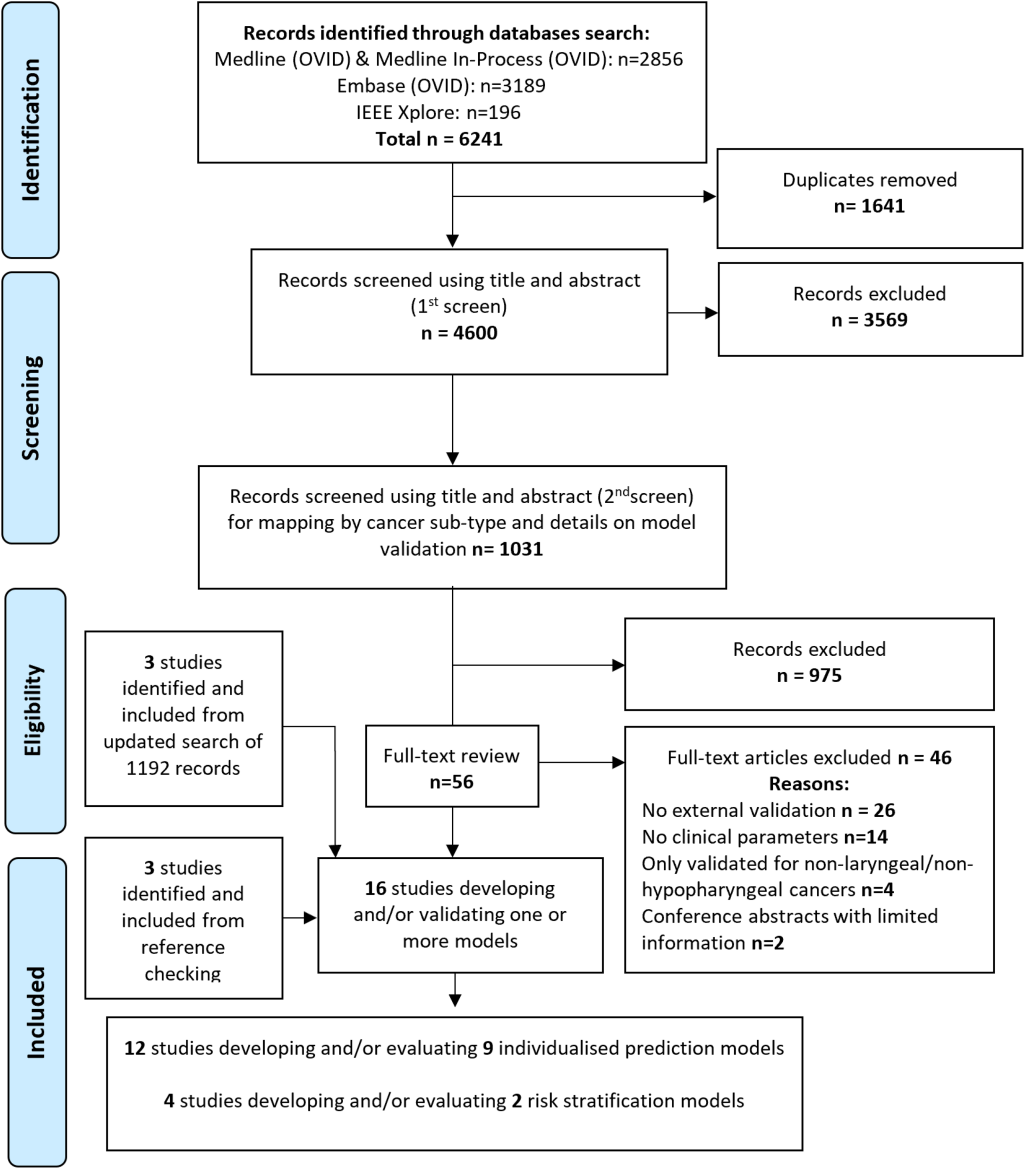
PRISMA flow diagram.

Sixteen studies, reporting the development and/or EV of nine individualized prediction models (IPMs), and two risk stratification models (RSMs), were included in this systematic review from 6241 articles identified on initial searches (**Figure 1**, **Table 2**).

**Table 2:** Models’ development and performance table.

Model	Study and year	Cohort	Location	Cohort size	Data source	Data collection period	Consecutive patients?	Outcomes predicted	Timing of measurement of model parameters	Model discrimination	Model calibration	Benchmarking to TNM system	Model development only	
													Algorithm	Type of predictors
IPM														
Chen	Chen 2021 (33)	Dev: Xiangya Hospital	China	95	LC	Retrospective (2009-2019)	NR	OS	Pre-treatment	Clinical & radiomic model: C-index for OS prediction = 0.78 (prediction horizon not specified)	2- and 3-year OS: calibration plots reported with good calibration	NR	LR	Clinical + Radiomic
		IV: Xiangya Hospital	China	41	LC	Retrospective (2009-2019)	NR	OS		Clinical & radiomic model: C-index for OS prediction = 0.75 (prediction horizon not specified)	2- and 3-year OS: calibration plots reported with good calibration	NR		
		EV: Hunan Cancer Hospital	China	54	LC	Retrospective (2011-2017)	NR	OS		Clinical & radiomic model: C-index for OS prediction = 0.75 (prediction horizon not specified)	2- and 3-year OS: calibration plots reported with good calibration	NR		
Datema	Datema 2013 (32)^a^	Dev: Leiden	Netherlands	1371	LC (ONCDOC)	Retrospective (1981-1998)	Yes	OS	Pre-treatment	OS: optimism-corrected C-index (based on 100 bootstrap samples) = 0.73 (prediction horizon not specified)	2- and 5-year OS: calibration plots reported	NR	CPH	Clinical
	Hoban 2017 (26)	EV: Michigan	United States	246	LC	Retrospective (2003-2014)	Likely yes	5-year OS		C-Index = 0.66, 5-year OS AUC (95% CI) = 0.68 (0.61-0.75)^c^	Calibration plots reported	NR		
Egelmeer	Egelmeer 2011 (24)	Dev: MAASTRO^d^	Netherlands	994	LC	Retrospective (1977-2008)	Yes	OS, LC	Pre-treatment	OS: AUC (95% CI) = 0.73 (0.70–0.77) LC: AUC (95% CI) = 0.67 (0.64–0.71) (prediction horizon not specified)	NR	Yes: AUC (95% CI) for OS = 0.62 (0.58–0.63), AUC (95% CI) for LC = 0.62 (0.55–0.63)	CPH	Clinical
		EV1: Leuven	Belgium	109	LC	Retrospective (2000-2006)	NR	OS, LC		OS: AUC (95% CI) = 0.68 (0.50–0.82), LC: AUC (95% CI) = 0.70 (0.50–0.78) (prediction horizon not specified)	NR	Yes: AUC (95% CI) for OS = 0.70 (0.45–0.81), AUC (95% CI) for LC = 0.62 (0.49–0.72)		
		EV2: VU	Netherlands	178	LC	Retrospective (2001-2007)	NR	OS, LC		OS: AUC (95% CI) = 0.74 (0.69–0.87), LC: AUC (95% CI) = 0.71 (0.66–0.81) (prediction horizon not specified)	NR	Yes: AUC (95% CI) for OS = 0.65 (0.57–0.75), AUC (95% CI) for LC = 0.64 (0.57–0.74)		
		EV3: NKI/AVL	Netherlands	205	LC	Retrospective (2000-2008)	NR	OS, LC		OS: AUC (95% CI) = 0.71 (0.60–0.82), LC: AUC (95% CI) = 0.62 (0.55–0.75) (prediction horizon not specified)	NR	Yes: AUC (95% CI) for OS = 0.57 (0.52–0.69), AUC (95% CI) for LC = 0.56 (0.49–0.63)		
		EV4: Manchester (1998-2005)	United Kingdom	403	LC	Retrospective (1998-2005)	NR	OS, LC		OS: AUC (95% CI) = 0.76 (0.72–0.81), LC: AUC (95% CI) = 0.72 (0.67–0.78) (prediction horizon not specified)	NR	Yes: AUC (95% CI) for OS = 0.63 (0.58–0.69), AUC (95% CI) for LC = 0.63 (0.58–0.69)		
	Hoban 2017 (26)	EV5: Michigan	United States	246	LC	Retrospective (2003-2014)	Likely yes	5-year OS		C-Index = 0.66, 5-year OS AUC (95% CI) = 0.72 (0.65-0.79)^c^	Calibration plots reported	NR		
	Aly 2021 (27)^,e^	EV6: NSW	Australia	105	LC	Retrospective (2010-2018)	NR	2-year OS, 2-year LR		2-year OS: AUC (95% CI) = 0.73 (0.61-0.85), 2-year LR: AUC (95% CI) = 0.59 (0.45-0.73)	Calibration plots reported with intercept and slope figures	NR		
	Ronn Hansen 2019 (28)^,e^	EV7: DAHANCA (2005-2015)^d^	Denmark	388	NCR (DAHANCA)	Retrospective (2005-2015)	NR	OS		OS: C-index (95% CI) = 0.78 (0.74-0.82) (prediction horizon not specified)	OS: calibration plots reported with very good calibration (2- year OS), but 5-year OS model underestimated survival	NR		
	Hansen 2022 (25)	EV8: Odnese/DAHANCA (2005-2018)^d^	Denmark	672	LC	Retrospective (2005-2018)	Yes	OS		OS: C-index (95% CI) = 0.74 (0.71-0.76) (prediction horizon not specified)	2- and 5-year OS: calibration plots reported	NR		
		EV9: Manchester (2005-2018)	United Kingdom	423	LC	Retrospective (2005-2018)	Yes	OS		OS: C-index (95% CI) = 0.70 (0.66-0.75) (prediction horizon not specified)	2- and 5-year OS: calibration plots reported	NR		
Emerick^b^	Emerick 2013 (34)^,a^	Dev: SEER	United States	50145	NCR (SEER)	Retrospective (1980-2009)	No	10-year CSM	Likely early post-treatment	C- index for predicting 10-years CSM percentiles (4% increments) = 0.99	10-years CSM: calibration plots reported	NR	Binary biological model: SNAP^f^	Clinical
	Hoban 2017 (26)	EV: Michigan	United States	246	LC	Retrospective (2003-2014)	Likely yes	5-year OS		C-Index = 0.68, 5-year OS AUC (95% CI) = 0.71 (0.64-0.78)^c^	10-years CSM: calibration plots reported	NR		
Lustberg	Lustberg 2016 (35)	Dev: MAASTRO^d^	Netherlands	978	LC	Retrospective (1977-2008)	Yes	2-year OS	Pre-treatment	2-year OS: AUC = 0.77, optimism-corrected AUC (based on 1000 bootstrap samples) =0.77 (+/- 2SD 0.73 - 0.81)	2-year OS: calibration plots underestimated survival for all groups especially the poor and medium prognosis ones	NR	CPH	Clinical
		EV1: Wollongong	Australia	Cohort with no Hb imputation: n=52 (Model with Hb imputation n=109)	LC (Uncurated automatically collected data)	Retrospective (1993-2012)	NR	2-year OS		2-year OS: AUC = 0.71, optimism-corrected AUC (based on 1000 bootstrap samples) = 0.72 (+/- 2SD 0.55 - 0.88), 2-year OS: AUC with low HB imputation = 0.72 (+/- 2SD 0.60 - 0.83), 2-year OS: AUC with high HB imputation = 0.73 (+/- 2SD 0.61 - 0.84), 2-year OS: AUC with median HB imputation = 0.74 (+/- 2SD 0.62 - 0.85)	2-year OS: calibration plots underestimated survival for all groups especially the poor and medium prognosis ones	NR		
		EV2: RTOG 91-11	United States	177	National RCT	Prospective (1992-2000)	No (RCT)	2-year OS		2-year OS: AUC = 0.57, optimism-corrected AUC (based on 1000 bootstrap samples) = 0.57 (+/- 2SD 0.47 - 0.67)	2-year OS: calibration plots underestimated survival for all groups especially the poor and medium prognosis ones, and overestimated survival for good prognosis group	NR		
Petersen	Petersen 2018 (29)	Dev: TNCR	Netherlands	3442	NCR(TNCR)	Retrospective (1991– 2010)	Likely no	OS	Pre-treatment	OS: C-index = 0.65 (prediction horizon not specified)	NR	Yes: C-index = 0.57	CPH	Clinical
		EV: Five pooled cohorts	Ireland/USA/Belgium/Sweden	770	Five independent LCs	Likely Retrospective data. Dates NR	NR	OS		OS: C-index = 0.59 (prediction horizon not specified)	Calibration plots reported	Yes: C-index = 0.55		
Tian	Tian 2021 (31)	Dev: SEER	United States	1758	NCR (SEER)	Retrospective (1975-2016)	Likely no	OS	Early post-treatment	OS: C-Index (95% CI) = 0.718 (0.709-0.727). Brier score = 0.179, 1-year OS: AUC = 0.748, 3-year OS: AUC = 0.741, 5-year OS: AUC = 0.731	Calibration plots reported	Yes: C-index (95% CI) = 0.627 (0.618-0.636), Brier score = 0.198, 1-year OS: AUC = 0.666, 3-year OS: AUC = 0.645, 5-year OS: AUC = 0.596	CPH	Clinical
		IV: SEER	United States	440	NCR (SEER)	Retrospective (1975-2016)	Likely no	OS		OS: C-Index (95% CI) = 0.708 (0.689-0.727). Brier score = 0.180, 1-year OS: AUC = 0.767, 3-year OS: AUC = 0.761, 5-year OS: AUC = 0.744	NR	Yes: C-index (95% CI) = 0.598 (0.578-0.617), Brier score = 0.201, 1-year OS: AUC = 0.651, 3-year OS: AUC = 0.668, 5-year OS: AUC = 0.582		
		EV: Fudan University	China	233	LC	Retrospective (2014-2017)	NR	OS		OS: C-Index (95% CI) = 0.709 (0.678-0.740). Brier score = 0.196, 1-year OS: AUC = 0.611, 3-year OS: AUC = 0.575, 5-year OS: AUC = 0.526	NR	Yes: C-index (95% CI) = 0.597 (0.570-0.624), Brier score = 0.225, 1-year OS: AUC = 0.639, 3-year OS: AUC = 0.650, 5-year OS: AUC = 0.513		
Zhu	Zhu 2020 (30)	Dev: SEER	United States	6,070	NCR (SEER)	Retrospective (2004-2015)	Likely no	5-year OS	Early post-treatment	5-year OS: C-index (95% CI) = 0.602 (0.592–0.612), optimism-corrected (based on 1000 bootstrap samples) C-index (95% CI) = 0.601 (0.595–0.607)	5-year OS: calibration plots reported	Yes: C-index (95% CI) = 0.547 (0.538–0.556)	CPH	Clinical
		EV: Fudan University	China	622	LC	Retrospective (2005-2010)	NR	5-year OS		5-year OS: C-index (95% CI) = 0.659 (0.594–0.724)	5-year OS: calibration plots reported	Yes: C-index (95% CI) = 0.608 (0.555–0.661)		
RSM														
Ho	Ho 2018 (37)	Dev: NCDB	United States	8351	NCR (NCDB)	Retrospective (2004-2013)	NR	3-year OS	Early post-treatment	3-year OS: C-index (95% CI) = 0.678 (0.665‐0.690), optimism‐corrected C-index (95% CI) = 0.674 (0.661‐0.687)	NR	Yes: 3-year OS C-Index (95% CI) = 0. 675 (0.663‐0.688), 3-year OS optimism‐corrected C-index (95% CI) = 0.671 (0.658‐0.684)	RPA	Clinical
	Choi 2019 (38)	EV: Seoul	Korea	141	LC	Retrospective (2006-2016)	Yes	3-year OS		3-year OS: C-index (95% CI) = 0.706 (0.601–0.811)	NR	NR		
Lacy	Lacy 1998 (40)	Dev: Missouri	United States	124	LC (two hospitals)	Retrospective (1980-1991)	NR	2-year OS	post-treatment (after tumour recurrence)	2-year OS after recurrence: C-index = 0.76	NR	NR	LR and conjunctive consolidation (clustering)	Clinical
	Lacy 2004 (39)	EV: Melbourne	Australia	67	LC	Retrospective (1989-1992)	NR	2-year OS		2-year OS after recurrence: C-index = 0.79	NR	Yes: 2-year OS after recurrence C-index = 0.63		

Dev, Development cohort; IV, internal validation; EV, external validation; IPM, Individualised prediction models; RSM, Risk stratification models; LC, Local cohort from one centre; NCR, National cancer registry; TNCR, The Netherlands cancer registry; NCDB, National cancer database (USA); SEER, Surveillance, Epidemiology, and End Results database (USA); DAHANCA, The Danish Head and Neck cancer Study Group database; RCT, Randomised controlled trial data; NR, Not Recorded; OS, Overall survival; CSM, Cancer-specific mortality; AUC, Area under ROC curve; CI, Confidence intervals, LR, Logistic regression; CPH, Cox proportional hazards model.

a Models developed using mixed head and neck cancer cohorts (not only LSCC/HPSCC).

b Based on the coefficients from Egelmeer 2011 model.

c 95% CI estimated.

d Potentially overlapping cohorts.

e Models reported in conference abstracts.

f SNAP model used g-values and complex mathematical equations.

### Individualized prediction models

#### Study characteristics

Main model and population characteristics are shown in **Supplementary Table 1B** and **Table 2**. The total number of patients included across all model development cohorts was 73,328, with a median cohort size of 1,371 (IQR 994-3,442). In the EV cohorts, the total number of patients was 5,394, with a median cohort size of 246 (IQR 177-418).

All of the studies included in this review were published between 2011 and 2021. Geographically, the development cohorts for all nine IPMs originated from the United States (four cohorts), the Netherlands (three cohorts), and China (one cohort) as shown in **Table 2**. Egelmeer 2011 model (24) had the broadest EV, being developed using a Dutch cohort and validated across nine cohorts from six countries: Belgium (24), the United Kingdom (UK) (24, 25), the United States (USA) (26), Australia (27), Denmark (25, 28), and the Netherlands (24). Similarly, Petersen 2018 model (29) had wide geographic EV, with development in a Dutch cohort and EV conducted on a pooled cohort from four countries (29): Ireland, the USA, Belgium, and Sweden. Both Zhu 2020 (30) and Tian 2021 (31) models were developed using data from the Surveillance, Epidemiology, and End Results (SEER) database in the United States but were externally validated only in Chinese cohorts. Similarly, the Datema 2013 model (32), developed on a Dutch cohort, was only externally validated in American patients (26). Models like Chen 2021 (33) and Emerick 2013 (34) had the most limited EV geographically, as these were validated only within the countries where they were originally developed (China and the USA, respectively).

The majority of development or EV cohorts (17/23, 74%) used retrospectively collected data obtained from single institutions (**Table 2**), except for the EV of the Egelmeer 2011 model by Ronn Hansen et al. (28 *)* that used data from the Danish Head and Neck Cancer Group (DAHANCA) national cancer registry, the Petersen 2018 development cohort, which was sourced from the Netherlands Cancer Registry (29), and the Zhu 2020, Tian 2021, and Emerick 2013 models, which were developed or validated using the SEER national database. Additionally, one model (Lustberg 2016) was externally validated using data from the Radiation Therapy Oncology Group (RTOG) 91-11 multi-centre randomised controlled trial (35).

Seven IPMs were developed and/or validated on patients with LSCC (24, 29, 30, 32, 34, 35), and two models [Chen 2021 (33) and Tian 2021 (31)] included only HPSCC patients. No models were found for non-squamous cell carcinoma histology. Two models (32, 34) were developed using mixed cohorts of laryngeal and non-laryngeal cancers, but were externally validated separately on LSCC/HPSCC patients (26).

**Figure 2:**
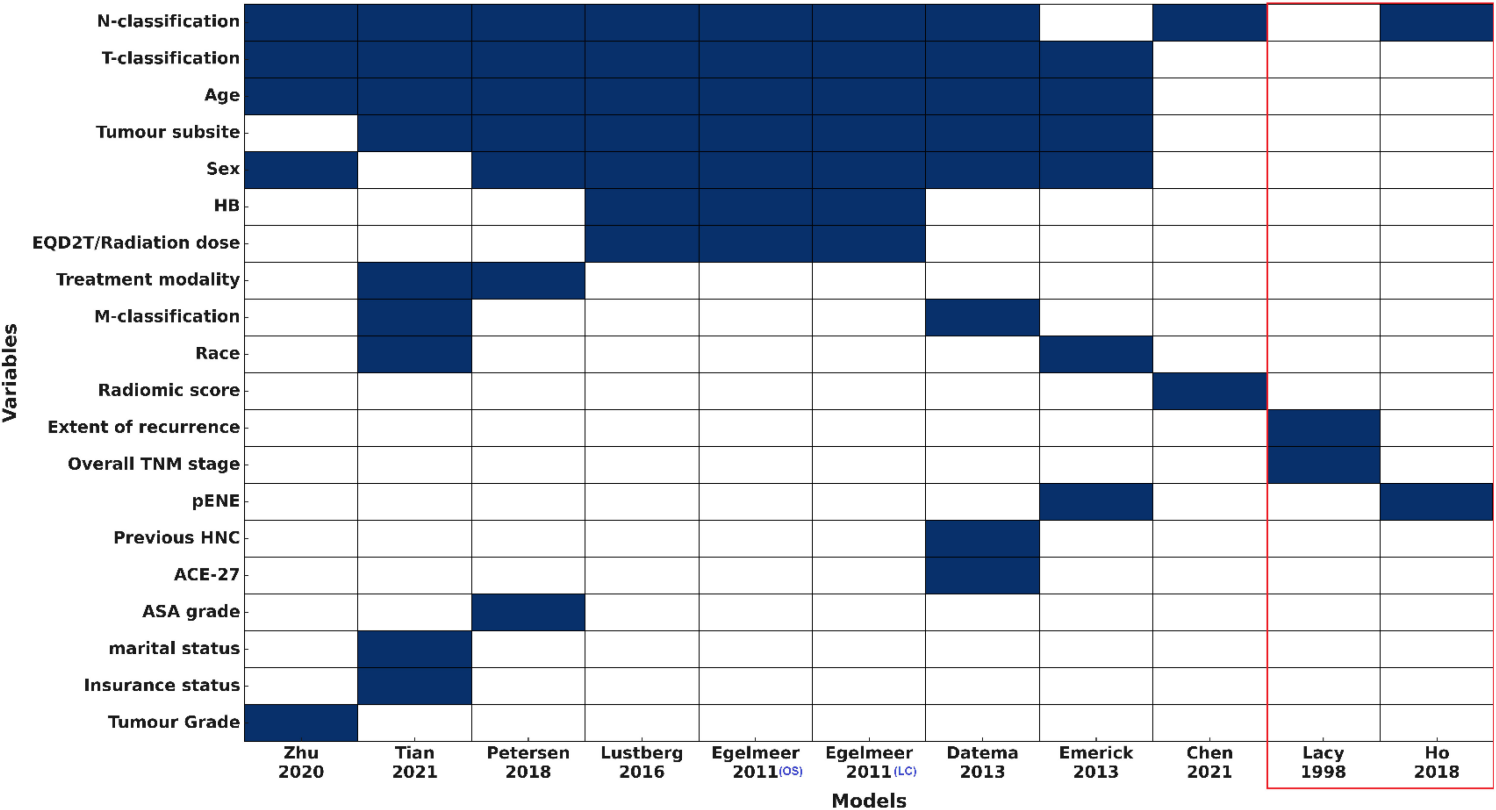
Predictors used for the included models (IPMs and RSMs) HB, haemoglobin level; pENE, extranodal extension on pathology; HNC, Head and Neck cancer; ACE-27, Adult Comorbidity Evaluation-27; ASA, The American Society of Anaesthesiologists physical status classification system. Variables are ranked by frequency of inclusion. Models within the red box represent the risk stratification models (RSMs).

Sex was reported for most cohorts (19/23, 82.6%), with males comprising the majority of patients (77%-98.3%). Smoking status was only reported in three studies (**Supplementary Table 1B**), but this variable was not incorporated in any of the included models (**Figure 2**). Only 5/23 cohorts (21.7%) reported TNM information (26, 31, 33), and TNM version was documented in just four cohorts across two IPMs. Despite this, N- and T-classifications were the most commonly used variables, included in 9/11 (81.8%) and 8/11 (72.7%) models, respectively.

Models included between two and nine variables each. Sociodemographic factors were variably represented: age in 8/11 (72.7%), sex in 7/11 (63.6%), and race, insurance status, and marital status in only one or two models. Comorbidity-related variables were rare, with Adult Comorbidity Evaluation-27 (ACE-27) and American Society of Anaesthesiologists (ASA) physical status were included in 1/11 (9.1%) and haemoglobin in 3/11 (27.3%) models. Treatment-related variables, including modality and radiation dose, along with tumour pathology factors like extranodal extension and tumour grade, were incorporated into one to three models each. All models included only clinical +/- histopathology variables, except the model by Chen et al., 2021 (33) that used clinical variables and a radiomics score. No models included clinical and any molecular/genomic variables (**Figure 2**).

Most patients in the included models were treated with curative intent, but there was variability in the proportion of patients receiving different treatment modalities across cohorts. Most cohorts (11/23, 47.8%) included patients treated only with definitive radiotherapy (RT) or chemoradiotherapy, while 8/23 (34.8%) cohorts included a mix of surgically treated and non-surgically treated patients, and only one cohort included exclusively surgically treated patients +/- RT (30).

Out of the nine IPMs included in our review, eight were developed using standard regression techniques: seven models employed Cox proportional hazards regression (24, 29–32, 35), and one model [Chen 2021 (33)] used logistic regression (**Table 2**). The exception was the Emerick 2013 (SNAP) model (34), which utilized g-values and complex mathematical equations.

Most models were externally validated only once except the Egelmeer 2011 OS and LC models (24), and the Lustberg 2016 OS model (24) that were externally validated in nine, five and two cohorts respectively (**Table 2** **).** Six models were independently validated in separate studies to the development ones using independent patient cohorts (TRIPOD type 4 validation (36)), and three models underwent EV within the same development study (TRIPOD type 3 validation (36)).

#### Model performance: overall survival

##### Development cohorts

**Figure 3:**
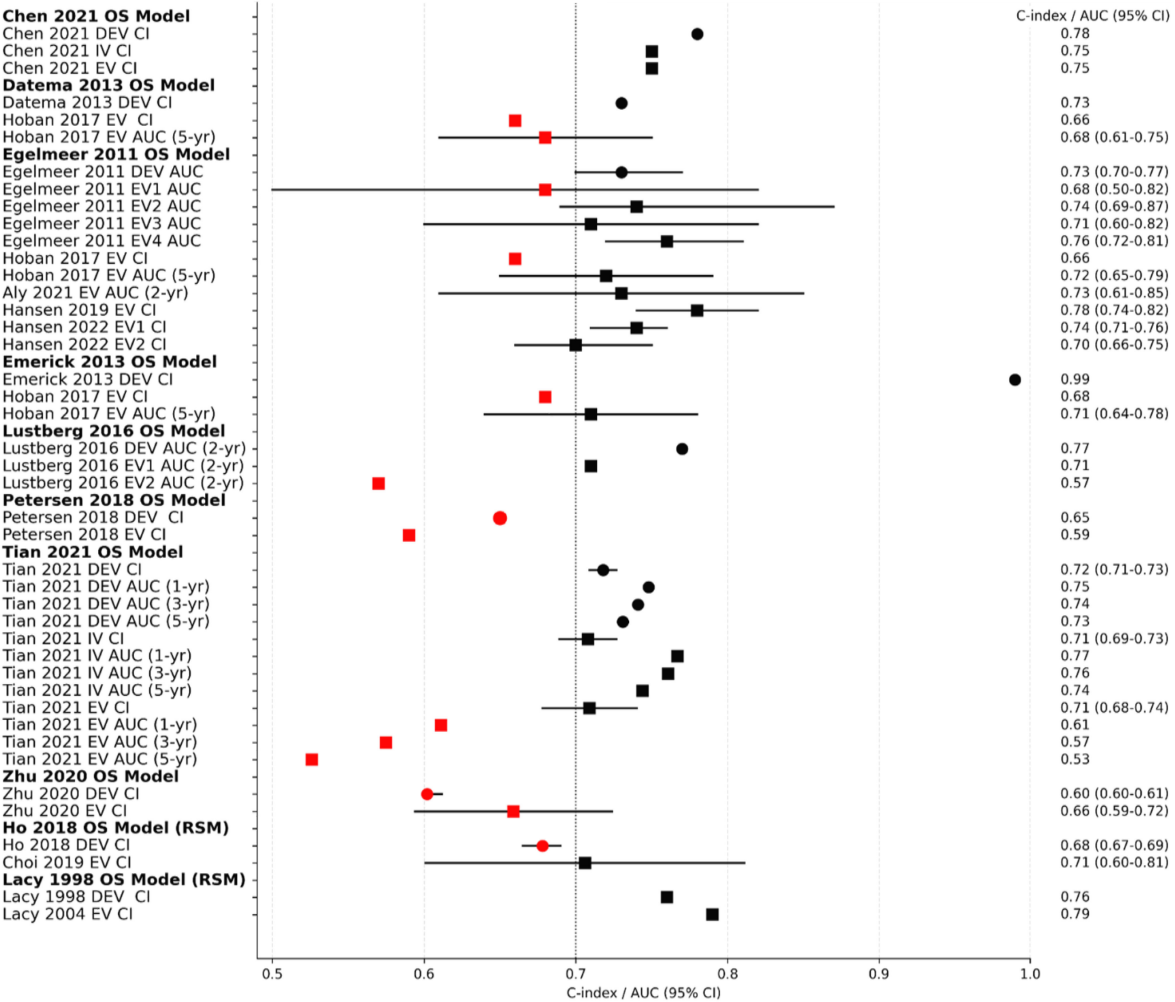
Overview forest plot for the C-Indices and/or AUCs of the overall survival models. Black dotted vertical line is a reference line set at AUC/C-index 0.7 (threshold between weak and good discrimination). Circles indicate models’ performance on development cohorts, while squares indicate performance on validation cohorts. Black squares/circles indicate good or excellent model discrimination, while red squares/circles indicate poor/weak discrimination. DEV; development cohort, IV; internal validation cohort, EV; external validation cohort, CI; C-Index, AUC; area under the curve, OS; overall survival, RSM; risk stratification model, 95% CI; 95% confidence interval.

Discrimination was ‘good’ for most of the model development studies and EVs, but only one model development had a C-index of >0.9 (excellent) (34). Five models (24, 26, 29, 30, 35) reported C-indices/AUCs < 0.7 indicating weak or poor discrimination, **Figure 3**. The discrimination performance of four models (24, 29–31) was superior to the TNM system when benchmarked against it, **Table 2**.

##### External validation cohorts

**Figure 4:**
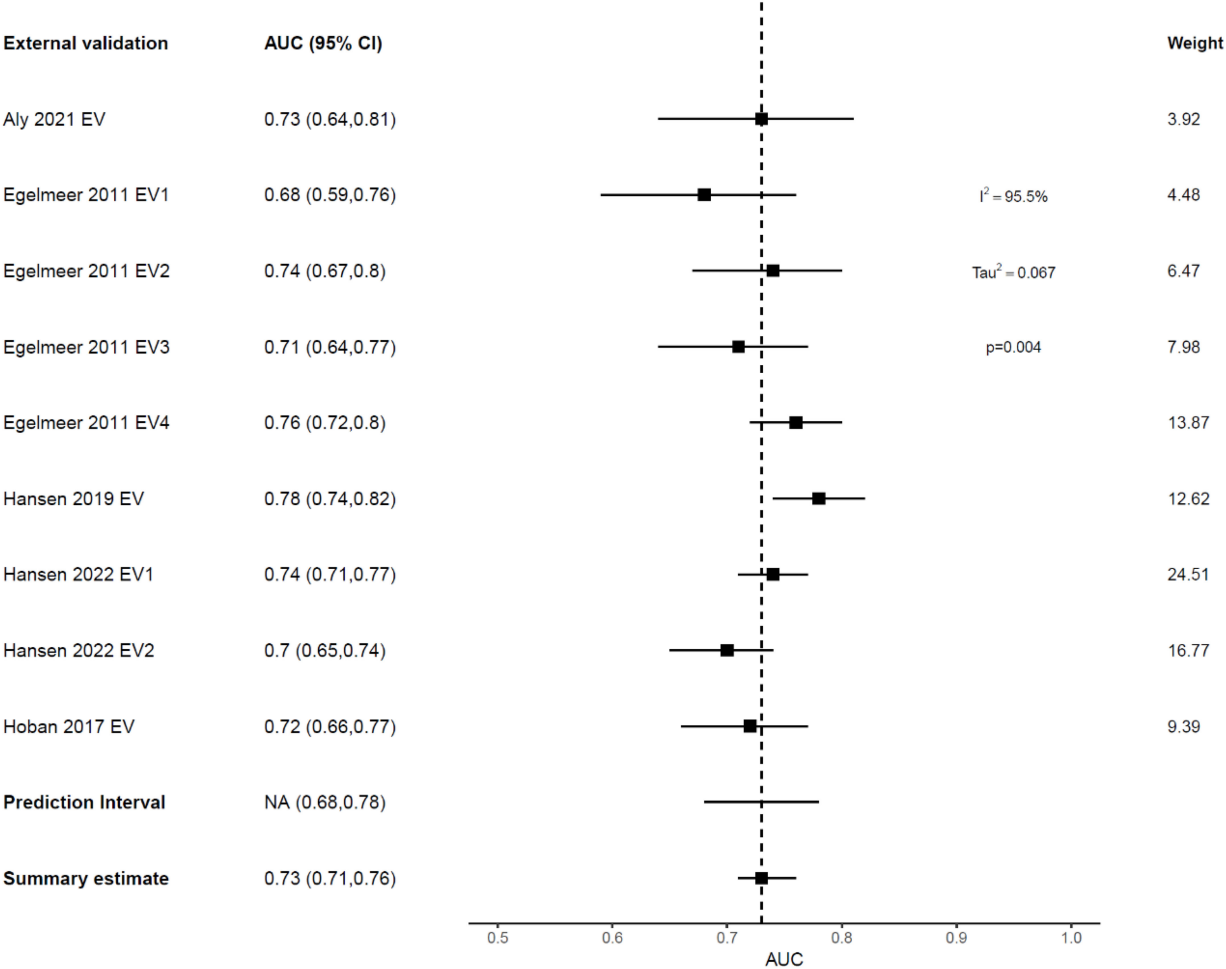
Forest plot showing the meta-analysis of the AUCs for all nine external validations of Egelmeer 2011 OS model. Black dotted vertical line is a reference line set at 0.73 to indicate the model’s discrimination in development study.

External validation showed deterioration in the discrimination ability of most models, indicating high variance and overfitting (**Figure 3**, **Table 3**). The Emerick 2013 (34) and Petersen 2018 (29) models had the highest variance and the least generalizability, as evidenced by dAUC% of -57% and dCI% of -40% respectively (**Table 3**). The Lustberg 2016 (35) model also suffered a large drop in discrimination performance on EV using the RTOG 91-11 cohort; dAUC% of -74.10%. The Chen 2021 (33) model was the only IPM that utilized a non-clinical variable (radiomics score), achieving a C-index of 0.78 in the derivation cohort, and 0.75 on EV (dCI% of -10.7%). External validations of the Egelmeer 2011 OS model (24) met the eligibility criteria for MA. The median dAUC% EV for this model based on nine EVs was 0% (no change), with IQR (−8.7% to 4.4%), **Table 3**. The pooled AUC for the nine EVs was 0.73 (95% CI 0.71-0.76), with an approximate 95% prediction interval of 0.68-0.78, **Figure 4**. As expected, the nine EV studies showed significant heterogeneity (*I* (2) = 95.5%).

**Table 3:** Absolute and percentage change in discrimination performance on external validation.

Model	Study and year	Cohort	Discrimination metric	Absolute Δ discrimination	% change in discrimination
Overall survival models					
Chen	Chen 2021 (33)	Dev: Xiangya Hospital	0.78		
		IV: Xiangya Hospital	0.75	-0.03	-10.70%
		EV: Hunan Cancer Hospital	0.75	-0.03	-10.70%
Datema	Datema 2013 (32)	Dev: Leiden	0.73		
	Hoban 2017 (26)	EV: Michigan	0.66	−0.07	−30.43%
Egelmeer	Egelmeer 2011 (24)	Dev: MAASTRO	0.73		
		EV1: Leuven	0.68	-0.05	-21.70%
		EV2: VU	0.74	0.01	4.40%
		EV3: NKI/AVL	0.71	-0.02	-8.70%
		EV4: Manchester (1998-2005)	0.76	0.03	13.00%
	Hoban 2017 (26)	EV5: Michigan	0.72	-0.01	-4.40%
	Aly 2021 (27)	EV6: NSW	0.73	0	0
	Hansen 2019 (28)	EV7: DAHNCA (2005-2015)	0.78	0.05	21.70%
	Hansen 2022 (25)	EV8: Odnese/DAHNCA (2005-2018)	0.74	0.01	4.40%
		EV9: Manchester (2005-2018)	0.7	-0.03	-13.00%
Emerick*	Emerick 2013 (34)	Dev: SEER	0.99		
	Hoban 2017 (26)	EV: Michigan	0.71	-0.28	-57.10%
Lustberg	Lustberg 2016 (35)	Dev: MAASTRO	0.77		
		EV1: Wollongong	0.71	-0.06	-22.20%
		EV2: RTOG 91-11	0.57	-0.2	-74.10%
Petersen	Petersen 2018 (29)	Dev: NCR	0.65		
		EV: Five pooled cohorts	0.59	−0.06	−40.00%
Tian	Tian 2021 (31)	Dev: SEER	0.718		
		IV: SEER	0.708	−0.01	−4.59%
		EV: Fudan University	0.709	−0.009	−4.13%
Zhu	Zhu 2020 (30)	Dev: SEER	0.602		
		EV: Fudan University	0.659	0.06	55.90%
Ho	Ho 2018 (37)	Dev: NCDB	0.678		
	Chen 2021 (33)	EV: Seoul	0.706	0.028	15.73%
Lacy	Lacy 1998 (40)	Dev: Missori	0.76		
	Lacy 2004 (39)	EV: Melbourne	0.79	0.03	11.50%
Local control/recurrence models					
Egelmeer	Egelmeer 2011 (24)	Dev: MAASTRO	0.67		
		EV1: Leuven	0.7	0.03	17.70%
		EV2: VU	0.71	0.04	23.50%
		EV3: NKI/AVL	0.62	-0.05	-29.40%
		EV4: Manchester (1998-2005)	0.72	0.05	29.40%
	Aly 2021 (27)	EV5: NSW	0.59	-0.08	-47.10%

Model calibration was reported for 16 model development/EVs, which showed good calibration for most models, but some underestimated OS (e.g., Lustberg 2016 (development and EV) (35), and Ronn Hansen 2019 (28) (EV for Egelmeer 2011). Brier scores were only reported for Tian 2021 model (31), which suggested borderline model performance (Brier score 0.2) in the EV cohorts (**Table 2**). MA of calibration performance was not possible for any model, as information on the total

number of observed (O) and expected (E) events were poorly reported (18, 19).

#### Model performance: tumor recurrence

**Figure 5:**
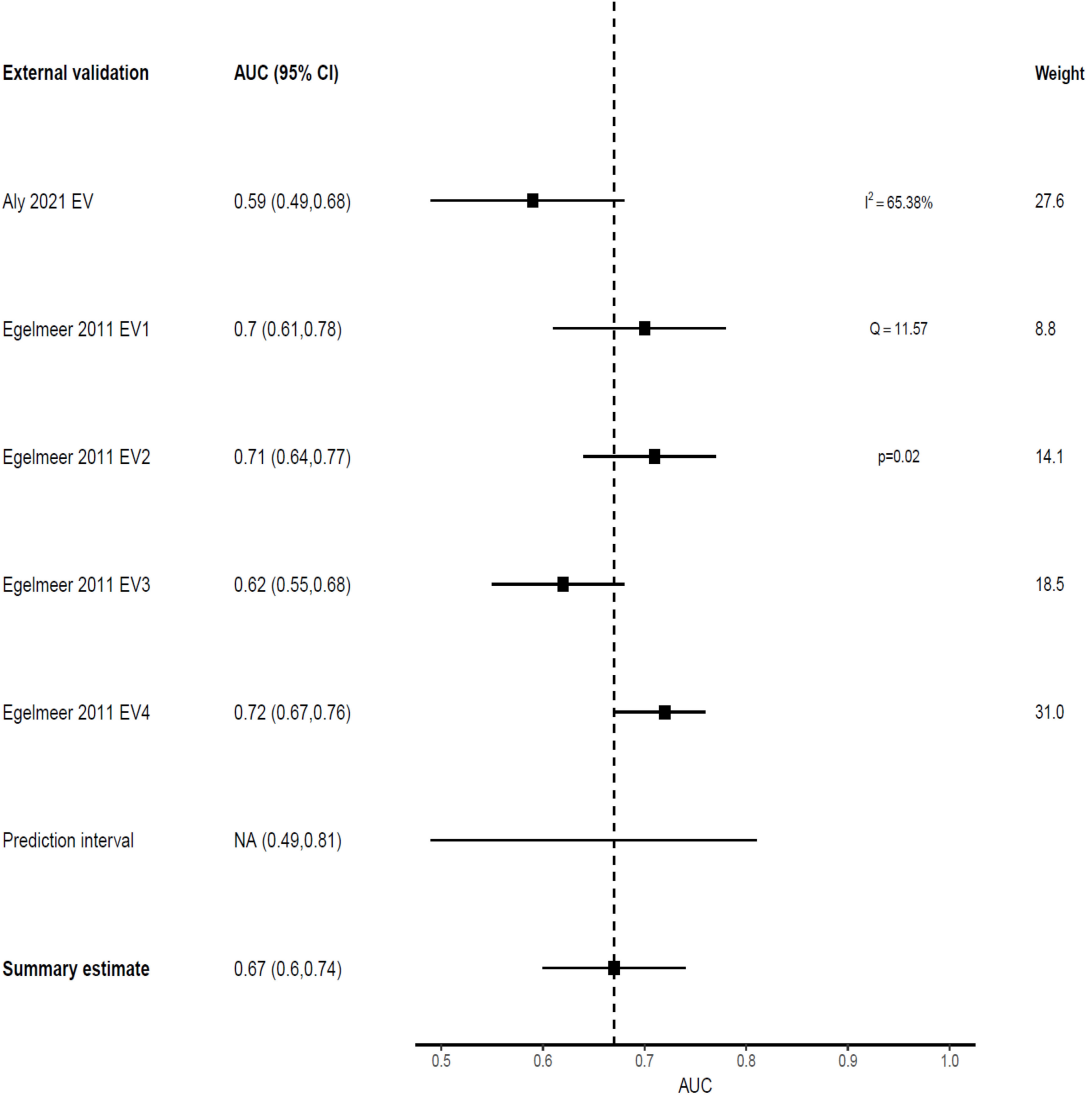
Forest plot showing the meta-analysis of the AUCs for all five external validations of Egelmeer 2011 LC model.

Only one model predicted non-survival outcomes; the Egelmeer 2011 local control (LC) model (24), that showed weak discrimination on model development with an AUC of 0.67 (95% CI 0.64–0.71). This model was externally validated in four cohorts within the same derivation study (TRIPOD type 3 validation). Additionally, this model was externally validated once by an independent team from Australia, that used local recurrence (LR) as the predicted outcome (27). The pooled AUC estimate for the five EVs was 0.67 (95% CI 0.6-0.74), with 95% prediction interval of 0.49-0.81, indicating only 34% probability of good discrimination on any future EV (**Figure 5**). However, when benchmarked against the TNM system, this model outperformed it in both the development cohort (TNM AUC 0.62) and EV cohorts (TNM AUC 0.56-0.64), **Table 2**.

### Risk stratification models: study characteristics and models’ performance

The SR only identified two RSMs (37–40), **Table 2** and **Supplementary Table 1B**. The Ho 2018 model (37) used recursive partitioning analysis on data from the American national cancer database, to modify the pathological N-stage and re-classify patients. All patients in that cohort were surgically-treated LSCC (84%) or HPSCC (16%). The model’s 3-year OS discrimination was weak with a C-index of 0.67 (95% CI 0.67‐0.69), which matched the performance of standard TNM-8 (C-index 0.67, 95% CI 0.66‐0.68). The model had one independent EV (38), and demonstrated a modest improvement in predictive performance (C-index 0.71, 95% CI 0.6–0.8, dCI% 15.7%).

Lacy et al. (40) used logistic regression and conjunctive consolidation to develop the Composite Laryngeal Recurrence Staging System (CLRSS), that they later externally validated in a separate study (39), **Table 2**. That model achieved good performance in predicting 2-year OS after LSCC recurrence in both the development cohort (C-index 0.76) and the EV cohort (C-index 0.79), compared to the TNM system (C-index 0.63).

### PROBAST risk of bias and applicability assessment

**Figure 6:**
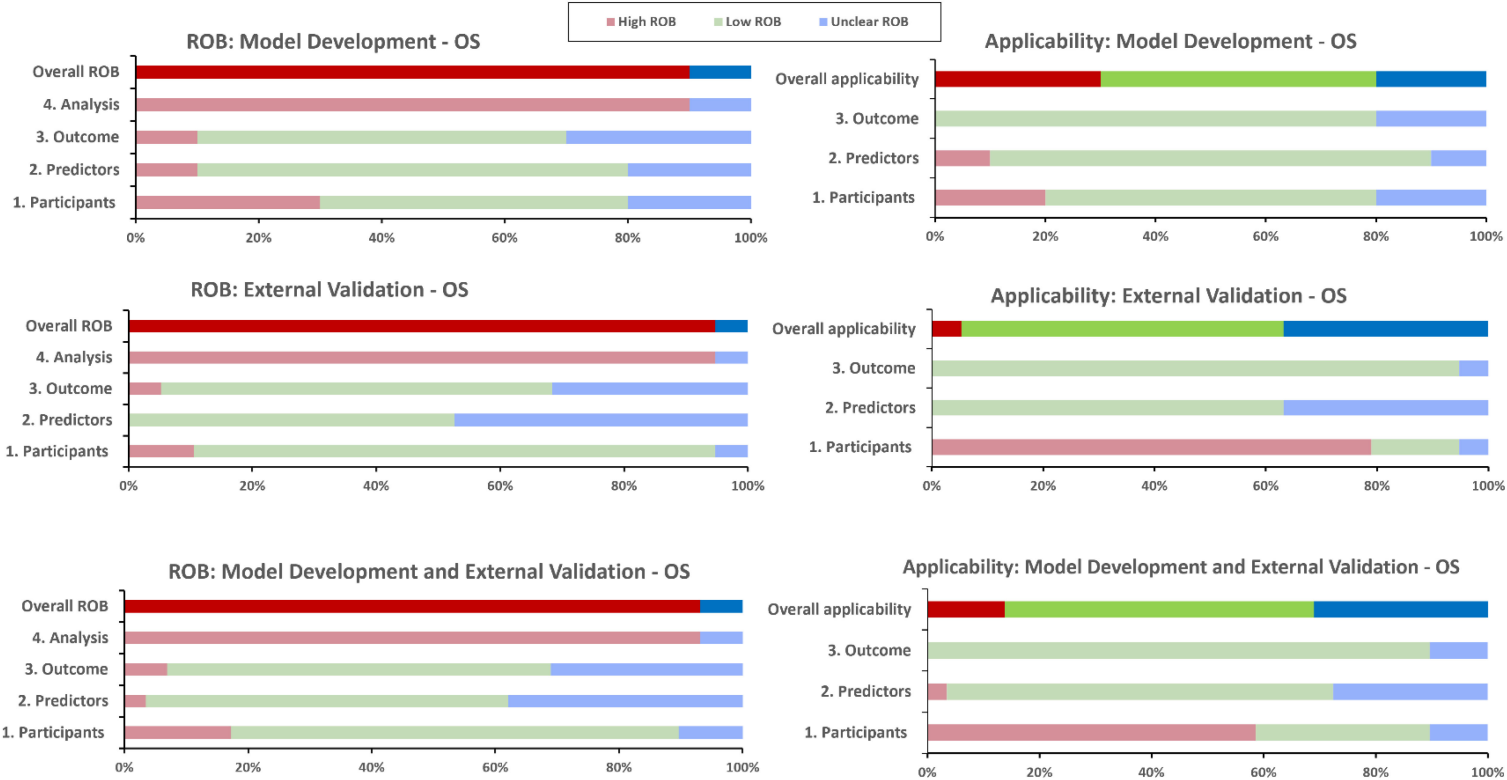
PROBAST risk of bias and applicability assessment for overall survival predictive models. OS, Overall survival; RoB, risk of bias.

The overall PROBAST RoB rating was high (or unknown) for the included models, and none achieved low RoB in the analysis domain (**Figure 6**, **Table 4**). Main areas of concern in the analysis domain included inappropriate handling of participants with missing data; selection of predictors based on univariate analysis; poor reporting of relevant model performance measures; unclear evidence that complexities in the data (such as competing risks) were accounted for; and difficulty in determining events per variable (EPV) metrics to check if cohorts had a reasonable number of participants. Zhu et al.’s model (30) excelled in EPV metrics, leveraging the expansive SEER registry for model development, and a substantial local cohort for validation. Similarly, the Egelmeer 2011 models (24) achieved satisfactory EPV, utilizing a large local cohort and a focused model consisting of seven variables.

**Table 4:** PROBAST risk of bias and applicability results for individual models.

Model	Study	Cohort	TRIPOD validation type* (36)	PROBAST risk of bias domains (ROB) N (%)					PROBAST applicability domains N (%)			
				Participants	Predictors	Outcome	Analysis	Overall	Participants	Predictors	Outcome	Overall
Overall survival: Individual prediction models for Laryngeal squamous cell carcinoma												
Datema 2013	Datema 2013 (32)	DEV: Leiden	/	?	+	+	?	?	?	+	+	?
	Hoban 2017 (26)	EV: Michigan	Type 4	+	+	?	–	–	+	+	+	+
Egelmeer 2011	Egelmeer 2011 (24)	DEV: MAASTRO	/	+	+	+	–	–	+	+	+	+
		EV1: Leuven	Type 3	+	?	+	–	–	+	?	+	?
		EV2: VU	Type 3	+	?	+	–	–	+	?	+	?
		EV3: NKI/AVL	Type 3	+	?	+	–	–	+	?	+	?
		EV4: Manchester (1998-2005)	Type 3	+	?	+	–	–	+	?	+	?
	Hoban 2017 (26)	EV5: Michigan	Type 4	+	+	?	–	–	+	+	+	+
	Aly 2021 (27)	EV6: NSW	Type 4	+	?	+	–	–	+	?	+	?
	Hansen 2019 (28)	EV7: DAHANCA (2005-2015)	Type 4	+	?	+	–	–	+	+	+	?
	Hansen 2022 (25)	EV8: Odnese/DAHANCA (2005-2018)	Type 4	+	?	+	?	?	+	+	+	+
		EV9: Manchester (2005-2018)	Type 4	+	?	+	–	–	+	?	+	+
Emerick 2013	Emerick 2013 (34)	DEV: SEER	/	–	?	?	–	–	–	?	?	–
	Hoban 2017 (26)	EV: Michigan	Type 4	+	+	?	–	–	+	+	+	+
Lustberg 2016	Lustberg 2016 (35)	DEV: MAASTRO	/	+	+	+	–	–	+	+	+	+
		EV1: Wollongong	Type 3	+	+	+	–	–	+	+	+	+
		EV2: RTOG 91-11	Type 3	+	+	+	–	–	+	+	+	+
Petersen 2018	Petersen 2018 (29)	DEV: TNCR	/	+	?	–	–	–	+	+	+	+
		EV: Five pooled cohorts	Type 3	+	?	–	–	–	+	+	+	+
Zhu 2020	Zhu 2020 (30)	DEV: SEER	/	?	+	+	–	–	?	+	+	?
		EV: Fudan University	Type 3	?	+	+	–	–	?	+	+	?
Overall survival: Individual prediction models for hypopharyngeal squamous cell carcinoma												
Chen 2021	Chen 2021 (33)	DEV: Xiangya Hospital	/	–	+	?	–	–	+	+	+	+
		IV: Xiangya Hospital	Type 2a	–	+	?	–	–	+	+	+	+
		EV: Hunan Cancer Hospital	Type 3	–	+	?	–	–	+	+	+	+
Tian 2021	Tian 2021 (31)	DEV: SEER	/	+	+	?	–	–	–	+	?	–
		IV: SEER	Type 2a	+	+	?	–	–	–	+	?	–
		EV: Fudan University	Type 3	+	+	?	–	–	–	+	?	–
Overall survival: Risk stratification models												
Ho 2018	Ho 2018 (37)	DEV: NCDB	/	–	–	+	–	–	+	–	+	–
	Choi 2019 (38)	EV: Korea	Type 4	–	+	?	–	–	+	+	+	+
Lacy 1998	Lacy 1998 (40)	DEV: Missouri	/	+	+	+	–	–	+	+	+	+
	Lacy 2004 (39)	EV: Melbourne	Type 4	+	+	+	–	–	+	+	+	+
Local recurrence/control model												
Egelmeer 2011	Egelmeer 2011 (24)	DEV: MAASTRO	/	+	+	+	–	–	+	+	+	+
		EV1: Leuven	Type 3	+	?	+	–	–	+	?	+	?
		EV2: VU	Type 3	+	?	+	–	–	+	?	+	?
		EV3: NKI/AVL	Type 3	+	?	+	–	–	+	?	+	?
		EV4: Manchester	Type 3	+	?	+	–	–	+	?	+	?
	Aly 2021 (27)	EV5: NSW	Type 4	+	?	+	–	–	+	?	+	?

Dev, Development cohort; IV, internal validation; EV, external validation; TNCR, The Netherlands cancer registry; NCDB, National cancer database (USA); SEER, Surveillance, Epidemiology, and End Results database (USA); DAHANCA, The Danish Head and Neck cancer Study Group database.

+ indicates low ROB/low concern regarding applicability, − indicates high ROB/high concern regarding applicability,? indicates unclear ROB/unclear concern regarding applicability.

* TRIPOD Type 2a validation (36): Internal validation by randomly splitting derivation cohort into 2 groups; TRIPOD Type 3 validation (36): External validation of a model’s performance using separate data (in the same study as model derivation); TRIPOD Type 4 validation (36): Independent external validation a model’s performance using separate data (in a separate study).

There were fewer concerns relating to the participants, predictors and outcomes RoB domains. However, only two models; Egelmeer 2011 (24) and Lustberg 2016 (35), had low RoB in all three non-analysis domains. In the predictors domain, there was universal lack of evidence that predictors were defined and assessed in similar way for all participants.

The development and validation cohorts for the included models appeared to be reasonably representative of unselected LSCC or HPSCC population. Most models included clinically-relevant predictors but they all failed to explain why other relevant predictors (e.g., smoking status), were not included. We also assessed the PROBAST applicability section to determine the extent to which the included IPMs match our SR question and PICOTS criteria (**Figure 6**, **Table 4**). The models were generally considered to have unclear or low concerns regarding applicability. However, two models, Tian 2021 (31) and Emerick 2013 (34), raised significant concerns about applicability, mainly due to difficulty in determining that palliative patients were excluded.

## Discussion

Outcome prediction models only have clinical transportability and statistical robustness if they can withstand performance assessment in multiple samples from the same or similar target populations (41). Despite the generation of numerous outcome prediction models for HNC, most remain confined to the development stage, or have shown poor external validation (8).

This study marks the first comprehensive SR of performance and RoB of externally-validated prognostic CPMs for LSCC and HPSCC patients. Our SR identified nine distinct IPMs and two RSMs. The SR by Aly et al. (8) only focused on assessing methodological quality and RoB using PROBAST (13) and the checklist for critical appraisal and data extraction for systematic reviews of prediction modelling studies (CHARMS) (14). Their review identified seven models for LSCC, with four overlapping in our SR, and three models that were only internally validated. The SR by Tham et al. (9) only focused on using the AJCC precision medicine core checklist (42) to evaluate prognostic nomograms for different HNCs, and only included three LSCC models.

Our findings indicate that available models for LSCC/HPSCC generally have good discrimination ability, especially on their development cohorts. Zhu 2020 (30) and Petersen 2018 (29) models showed weak performance in the development cohorts and on EV. Interestingly, these models were developed using data from population-based cancer registries (SEER and NCR respectively), which, despite their extensive case numbers, have been criticized for including potentially heterogeneous data (43, 44). Moreover, reliance on pre-collected datasets, such as trial data or national registries, presents a persistent problem for CPM: their inflexibility due to preset variables, which may not include all relevant predictors for the cancer being modelled, and potentially leading to poor performing models. Furthermore, population-based registries are notorious for containing significant data gaps, necessitating effective imputation strategies to preserve valuable information (45). The decision by Zhu et al. (30) and Petersen et al. (29) to exclude cases with missing data may have inadvertently contributed to the poor performance of their models, especially during the development phase.

Most of the variables used in LSCC/HPSCC prognostic models reflected solid underlying biological or pathological mechanisms linked to patient outcomes. Advanced T- and/or N-classification, for instance, is linked to higher recurrence rates and poorer survival (46, 47). Tumour grade and extranodal extension are also important predictors of aggressive disease and worse prognosis (38, 48, 49). Haemoglobin level is also a potentially crucial predictor, particularly in LSCC/HPSCC models for patients treated with RT, as low haemoglobin leads to tumour hypoxia, making the cancer less responsive to treatment and decreasing survival (50–52). Sociodemographic factors like age and sex impact prognosis, with older patients and males facing worse outcomes due to greater exposure to risk factors like smoking and reduced treatment tolerance (53, 54). Moreover, race and insurance status further affect survival, as disparities in healthcare access can lead to delayed diagnosis and treatment (53, 55, 56).

While these important predictors such as N- and T-classifications, sex and tumour subsite, were frequently included, it’s notable that known significant risk factors like smoking status were rarely incorporated. This highlights a significant gap in the predictive modelling landscape for LSCC/HPSCC patients, with continued smoking after completion of treatment being well-established as an independent risk factor for survival in those patients (57–60). Moreover, heterogeneity in treatment modalities across different cohorts may influence model predictions and applicability in diverse clinical contexts. For instance, in the Chen 2021 (33) model, surgical treatment dominated the development cohort (85%), whereas in other cohorts, such as the Egelmeer 2011 (24), Tian 2021 (31) and Lustberg 2016 (35) models development cohorts, there was significant proportions undergoing radiotherapy-only treatments. These aspects warrant consideration, especially when tailoring treatment strategies based on model predictions.

The selection criteria of the training population are vital to the quality and generalisability of predictive models. In cohorts focused on specific treatments like RT or chemoradiation [e.g., Petersen 2018 (29) and Egelmeer 2011 (24)], rigorous patient selection is essential for ensuring valid predictions. While narrow selection may enhance model performance within that specific group, it limits broader applicability. For example, models based on Egelmeer’s cohort, where 100% of patients received radiotherapy-only, may not generalise well to patients undergoing primary surgical treatment. This underscores the need for clinicians to carefully consider the development and validation cohorts when assessing a model’s applicability to their own patient populations.

On external validation, a discernible trend of decreased model performance was observed universally, indicating possible overfitting and/or a mismatch between training and validation populations. These issues are detrimental for cancer outcome prediction with excessively tailored models performing well on training data but failing to generalize well to new unseen data and therefore not useful in a clinical setting (41, 61). A good example is the Emerick 2013 model (34), that was trained on a dataset from the SEER registry encompassing over 50,000 cases. That model demonstrated excellent performance for predicting 10-year cancer specific mortality in the development cohort (C-index 0.99), but failed to adequately generalize on a local cohort (C-index 0.68). Interestingly, Tian 2021 model (31) that also trained on SEER HPSCC data, also failed to externally validate on a local cohort from China. Another notable finding is that the models trained on mixed HNC cohorts, e.g., Emerick 2013 (34) and Datema 2013 (32), both showed weak discrimination on EV using datasets specific to LSCC/HPSCC patients.

Two models, Egelmeer 2011 (24) and Chen 2021 (33), demonstrated promising performance in both development and EV. However, caution is advised since none achieved ‘very good’ or ‘excellent’ discrimination that would allow them to be recommended for routine clinical application. The Egelmeer 2011 (24) model was the most externally validated model with four TRIPOD type 3 EVs; AUCs 0.68-0.76, and five TRIPOD type 4 independent EVs; AUCs 0.66-0.78. TRIPOD type 4 validation, using entirely independent cohorts from varied settings or populations, is seen as a robust EV due to its stringent process (61–63). Moreover, this model was developed using a well-defined cohort of 994 consecutive LSCC patients, all treated with radiotherapy, with a relatively long median follow-up of 72 months. Egelmeer et al. (24) also applied strict inclusion and exclusion criteria, clearly defined the predicted outcomes, and managed missing data using predictive mean matching. They also employing bootstrapping to adjust for optimism, with a sufficient events-per-variable ratio of approximately 20.

Interestingly, the model by Chen et al. (33) was trained and externally validated on relatively small datasets; 95 and 54 HPSCC cases respectively. Despite the limited data, it exhibited reasonable performance in both the development cohort (C-index 0.78) and EV (C-index 0.75). This was the only model that integrated a robust radiomic score, derived from six radiomic features, alongside a single clinical predictor (N-classification). This model had only one EV in the same derivation study, and no independent TRIPOD type 4 EV, so generalizability cannot be fully determined. However, radiomic features and scores in HNC are gaining recognition for improving predictive accuracy, but their reproducibility and practicality in routine clinical settings require careful consideration (64, 65).

The models in our SR have overwhelmingly demonstrated high RoB across all domains. Future modeling efforts should pay special attention to the analysis domain, as this was most frequently rated as having a high RoB in our SR and across the literature (66, 67). Addressing bias is critical to ensure accurate predictions, which requires careful consideration of data collection, preprocessing, and algorithm design. Furthermore, reducing bias can also potentially enhance models’ performance and generalizability across diverse cohorts (23).

Moreover, the current predictive models for LSCC and HPSCC are further limited by poor external validation and the exclusion of critical predictors like smoking status, which can significantly impact outcomes. Additionally, inconsistent reporting of key variables, such as TNM staging, limits the ability to fully assess model performance. These issues, along with the high risk of bias observed in many models, reduce their generalizability across diverse patient populations and treatment modalities. Addressing these limitations, alongside improvements in bias reduction, is essential for future models to be more robust and applicable in clinical practice. By incorporating comprehensive predictor sets and ensuring more inclusive external validation, future efforts can improve the utility and performance of predictive models in varied clinical settings.

The strengths of this SR-MA include adhering to the TRIPOD-SRMA and CHARMs guidelines, and used the PROBAST ROB tool. Moreover, we used a comprehensive literature search strategy that incorporated the IEEE database, along with EMBASE and MEDLINE databases, resulting in the retrieval of 4,600 unique titles. However, we acknowledge that incorporating additional databases such as Scopus and/or Web of Science could potentially have further improved both the recall and precision of our search strategy. We also performed quantitative analysis when feasible, using robust methods for pooling summary estimates. The limitations of this SR-MA include the exclusion of models based solely on radiomic or genomic features that did not include clinical variables. Although these may significantly influence future modeling for LSCC/HPSCC, their current clinical utility remains unknown. Moreover, models that were not externally validated were excluded, with the possibility that these demonstrate good performance in future validations. It’s also important to acknowledge that the discrimination thresholds we used for AUCs/C-indices were arbitrarily set, and might vary based on models’ application and the prevalence of outcomes.

## Conclusion

While the integration of real-world data into CPMs holds promise, our review highlights the necessity of rigorous evaluation for their effective and safe use in routine practice. Despite the proliferation of models, most exhibited methodological limitations and bias, underscoring the need for careful scrutiny. Currently, no models can confidently be recommended for routine clinical use. Clinicians should choose LSCC and HPSCC prognostic models that match their patients’ characteristics, and have undergone thorough external validation ideally involving a local cohort. Future modelling efforts for LSCC/HPSCC should incorporate clinically important candidate predictors, and explore the addition of radiomic and/or biomolecular predictors for each treatment setting to potentially enhance performance.

## Data Availability

The original contributions presented in the study are included in the article/**Supplementary Material**. Further inquiries can be directed to the corresponding author.

## PETNECK2 Research Team

Ahmad Abou-Foul (Institute of Head and Neck Studies and Education, Department of Cancer and Genomic Sciences, University of Birmingham, University of Birmingham) A.Abou-Foul@bham.ac.uk; Andreas Karwath (Department of Cancer and Genomic Sciences, University of Birmingham) A.Karwath@bham.ac.uk; Ava Lorenc (QuinteT research group, Bristol Medical School, University of Bristol) ava.lorenc@bristol.ac.uk; Barry Main (University Hospitals Bristol and Weston NHS Trust) B.G.Main@bristol.ac.uk; Claire Gaunt (Cancer Research UK Clinical Trials Unit, University of Birmingham) C.H.Gaunt@bham.ac.uk; Colin Greaves (School of Sport, Exercise and Rehabilitation Sciences, University of Birmingham) C.J.Greaves@bham.ac.uk; David Moore (Department of Applied Health Research, University of Birmingham) D.J.MOORE@bham.ac.uk; Denis Secher (Patient Representative); Eila Watson (Oxford School of Nursing and Midwifery, Oxford Brookes University) ewatson@brookes.ac.uk; Georgios Gkoutos (Department of Cancer and Genomic Sciences, University of Birmingham) G.Gkoutos@bham.ac.uk; Gozde Ozakinci (Health Psychology Research Group, University of Stirling) Go7@stir.ac.uk; Hisham Mehanna (Institute of Head and Neck Studies and Education, Department of Cancer and Genomic Sciences, University of Birmingham) h.mehanna@bham.ac.uk; Jane Wolstenholme (Nuffield Department of Population Health, University of Oxford) jane.wolstenholme@dph.ox.ac.uk; Janine Dretzke (Department of Applied Health Research, University of Birmingham) J.Dretzke@bham.ac.uk; Jo Brett (Department of Midwifery, Community and Public Health, Oxford Brookes University) jbrett@brookes.ac.uk; Joan Duda (School of Sport, Exercise and Rehabilitation Sciences, University of Birmingham) J.L.DUDA@bham.ac.uk; Julia Sissons (Cancer Research UK Clinical Trials Unit, University of Birmingham) j.a.sissons@bham.ac.uk; Lauren Matheson (Oxford Institute of Nursing, Midwifery and Allied Health Research, Oxford Brookes University) l.matheson@brookes.ac.uk; Lucy Speechley (Cancer Research UK Clinical Trials Unit, University of Birmingham) L.J.Speechley@bham.ac.uk; Marcus Jepson (QuinteT research group, Bristol Medical School, University of Bristol) Marcus.jepson@bristol.ac.uk; Mary Wells (Nursing Directorate, Imperial College Healthcare NHS Trust, Charing Cross Hospital) mary.wells5@nhs.net; Melanie Calvert (Institute of Applied Health Research, University of Birmingham) M.Calvert@bham.ac.uk; Pat Rhodes (Patient Representative); Paul Nankivell (Institute of Head and Neck Studies and Education, Department of Cancer and Genomic Sciences, University of Birmingham) p.c.nankivell@bham.ac.uk; Philip Kiely (University Hospitals Bristol and Weston NHS Foundation Trust) Philip.Kiely@UHBristol.nhs.uk; Piers Gaunt (Cancer Research UK Clinical Trials Unit, University of Birmingham) P.Gaunt@bham.ac.uk; Saloni Mittal (Institute of Head and Neck Studies and Education, Department of Cancer and Genomic Sciences, University of Birmingham) s.mittal.2@bham.ac.uk; Steve Thomas (Bristol Dental School, University of Bristol) Steve.Thomas@bristol.ac.uk; Stuart Winter (Nuffield Department of Surgical Sciences, University of Oxford) Stuart.Winter@ouh.nhs.uk; Tessa Fulton-Lieuw (Institute of Head and Neck Studies and Education, Department of Cancer and Genomic Sciences, University of Birmingham) m.t.fulton-lieuw@bham.ac.uk; Wai-lup Wong (East and North Hertfordshire NHS Trust, Mount Vernon Cancer Centre) wailup.wong@nhs.net; Yolande Jefferson (Cancer Research UK Clinical Trials Unit, University of Birmingham) y.c.jefferson@bham.ac.uk.
