# Supplementary material for: Clinical predictive models for recurrence and survival in treated laryngeal and hypopharyngeal cancer: a systematic review and meta-analysis
Source: Front Oncol. 2024 Dec 6;14:1478385. doi: 10.3389/fonc.2024.1478385 (PMC11659268; doi:10.3389/fonc.2024.1478385)
Supplement: Supplementary file 1 [file Table1.docx]

**Supplementary Table 1A. Search strategy**

| **Embase**  1 (head adj2 neck adj (cancer$ or carcinoma$ or neoplasm$ or mass$ or tumo?r$ or adenoma$ or lesion$ or metasta$ or malignan$ or nodule$)).ti,ab.  2 exp "head and neck tumor"/  3 ((oral cavity or pharyn$ or nasopharyn$ or oropharyn$ or hypopharyn$ or larynx or laryngeal or paranasal sinus$ or salivary gland or nasal cavity or oral cavity or tongue or lip or gingival or facial or mouth or tracheal or otorhinolaryngological) adj2 (cancer$ or carcinoma$ or neoplasm$ or mass$ or tumo?r$ or adenoma$ or lesion$ or metasta$ or malignan$ or nodule$)).ti,ab  4 (c-index or concordance index or c-statistic or concordance statistic or Nomogram$ or (predict$ adj3 model$) or (prognos$ adj3 model$) or (calibrat$ adj3 model$) or (discriminat$ adj3 model$) or Area under the curve or AUC or (Validat$ and (cohort$ or set$ or internal or external))).ti,ab.  5 1 or 2 or 3  6 exp cancer survival/  7 exp progression free survival/  8 exp overall survival/  9 exp cancer prognosis/  10 exp cancer recurrence/  11 exp disease free survival/  12 ((cancer$ or carcinoma$ or neoplasm$ or mass$ or tumo?r$ or adenoma$ or lesion$ or metasta$ or malignan$ or nodule$) adj5 (pfs or dfs or os or "progression free survival" or "disease free survival" or "overall survival")).ti,ab.  13 ((cancer$ or carcinoma$ or neoplasm$ or mass$ or tumo?r$ or adenoma$ or lesion$ or metasta$ or malignan$ or nodule$) adj5 (recur$ or relaps$ or regre$ or metastas$ or progres*)).ti,ab.  14  6 or 7 or 8 or 9 or 10 or 11 or 12 or 13  15 4 and 5 and 14  16 limit 15 to yr="2005 -Current" (3189) |
| --- |
| **MEDLINE and In-Process**  1 (head adj2 neck adj (cancer$ or carcinoma$ or neoplasm$ or mass$ or tumo?r$ or adenoma$ or lesion$ or metasta$ or malignan$ or nodule$)).ti,ab.  2 exp "head and neck neoplasms"/  3 ((oral cavity or pharyn$ or nasopharyn$ or oropharyn$ or hypopharyn$ or larynx or laryngeal or paranasal sinus$ or salivary gland or nasal cavity or oral cavity or tongue or lip or gingival or facial or mouth or tracheal or otorhinolaryngological) adj2 (cancer$ or carcinoma$ or neoplasm$ or mass$ or tumo?r$ or adenoma$ or lesion$ or metasta$ or malignan$ or nodule$)).ti,ab.  4 (c-index or concordance index or c-statistic or concordance statistic or Nomogram? or nomogram, or (predict$ adj3 model$) or (prognos$ adj3 model$) or (calibrat$ adj3 model$) or (discriminat$ adj3 model$) or Area under the curve or AUC or (Validat$ and (cohort$ or set$ or internal or external))).ti,ab.  5 1 or 2 or 3  6 exp cancer survivors/  7 exp progression-free survival/  8 exp survival/  9 exp prognosis/  10 exp neoplasm recurrence, local/  11 exp disease-free survival/  12 ((cancer$ or carcinoma$ or neoplasm$ or mass$ or tumo?r$ or adenoma$ or lesion$ or metasta$ or malignan$ or nodule$) adj5 (pfs or dfs or os or "progression free survival" or "disease free survival" or "overall survival")).ti,ab.  13 ((cancer$ or carcinoma$ or neoplasm$ or mass$ or tumo?r$ or adenoma$ or lesion$ or metasta$ or malignan$ or nodule$) adj5 (recur$ or relaps$ or regre$ or metastas$ or progres*)).ti,ab.  14 6 or 7 or 8 or 9 or 10 or 11 or 12 or 13  15 4 and 5 and 14  16 limit 15 to yr="2005 -Current" (2856) |
| **IEEE database**  **Search 1**  "head and neck" OR "oral cavity" OR pharyn* OR nasopharyn* OR hypopharyng*  AND  cancer OR tumour* OR tumour* OR carcinoma* OR metastas* OR malignancies OR malignancy  AND  model OR "c-index" OR "c-statistic" OR nomogram*  **Search 2**  larynx OR laryngeal OR "paranasal sinus*" OR "salivary gland" OR "nasal cavity" OR "oral cavity" OR tongue OR lip OR gingiva OR facial OR mouth OR tracheal OR otorhinolaryngological  AND  cancer OR tumour* OR tumour* OR carcinoma* OR metastas* OR malignancies OR malignancy  AND  model OR "c-index" OR "c-statistic" OR nomogram*  **Search 3**  larynx OR laryngeal OR "paranasal sinus*" OR "salivary gland" OR "nasal cavity" OR "oral cavity" OR tongue OR lip OR gingiva OR facial OR mouth OR tracheal OR otorhinolaryngological  AND  neoplasm OR mass OR nodule OR adenoma OR lesion  AND  "concordance index" OR "area under the curve" OR calibration OR validation  **Search 4**  "head and neck" OR "oral cavity" OR pharyn* OR nasopharyn* OR hypopharyng*  AND  neoplasm OR mass OR nodule OR adenoma OR lesion  AND  "concordance index" OR "area under the curve" OR calibration OR validation |

**Supplementary Table 1B. Clinical and population characteristics of included models**

| **Clinical characteristics table** | | | | | | | | | | | | | |
| --- | --- | --- | --- | --- | --- | --- | --- | --- | --- | --- | --- | --- | --- |
| **Model** | **Study** | **Cohort** | **Tumour location** | **Age (mean (SD), years)** | **Males n (%)** | **TNM version and stage** | **T,N,M information** | **Smokers, n (%)** | **Alcohol consumption, n (%)** | **Cancer treatment** | **Co-morbidities** | **Follow-up duration** | **Number (proportion) of events** |
| **IPM** | | | | | | | | | | | | | |
| **Chen** | **Chen 2021** | Dev: Xiangya Hospital | HPSCC | 58.7 | NR | TNM-8: I n=0; II n=16 (16.8%); III n=22 (23.2%); IV n=57 (60%) | T1+T2 n=33 (34.7%); T3+T4 n=62 (65.3%). N0 n=30 (31.6%), N1 n=19 (20%), N2 n=38 (40%), N3 n=8 (8.4%). All M0 | n=70 (73.7%) | n=63 (66.3%) | Surgery n=81 (85.3%), non-surgical treatment n=14 (14.7%) | NR | NR (range 1-120 months) | NR |
|  |  | IV: Xiangya Hospital | HPSCC | 59.3 | NR | TNM-8: I n=0; II n=5 (12.2%); III n=12 (29.3%); IV n=24 (58.5%) | T1+T2 n=12 (29.3%); T3+T4 n=29 (70.7%). N0 n=13 (31.7%), N1 n=10 (24.4%), N2 n=16 (39%), N3 n=2 (4.9%). All M0 | n=28 (68.3%) | n=26 (63.4%) | Surgery n=33 (80.5%), non-surgical treatment n=8 (19.5%) | NR | NR | NR |
|  |  | EV: Hunan Cancer Hospital | HPSCC | 58.7 | NR | TNM-8: I n=0; II n=4 (7.4%); III n=12 (22.2%); IV n=38 (70.4%) | T1+T2 n=24 (44.4%); T3+T4 n=30 (55.6%). N0 n=9 (16.7%), N1 n=13 (24.1%), N2 n=30 (55.6%), N3 n=2 (3.7%). All M0 | n=47 (87%) | n=39 (72.2%) | Surgery n=20 (37%, non-surgical treatment n=34 (63%) | NR | NR | NR |
| **Datema*** | **Datema 2013^%^** | Dev: Leiden | SCC originating from lip, Oral cavity, Oropharynx, Nasopharynx, Hypopharynx, Larynx-glottic/supraglottic | 62.6 (12.0) | 1088 (79.4%) | NR | T1 n=516 (37.6%); T2 n=369 (26.9%); T3 n=208 (15.2%); T4 n=278 (20.3%). N0 n=964 (70.3%); N1 n=145 (10.6%); N2 n=180 (13.1%); N3 n=82 (6.0%). M0 n=1354 (98.8%); M1 n=17 (1.2%) | NR | NR | NR | ACE-27: Grade 0 (none) n=845 (61.6%); Grade 1 (mild) n=251 (18.3%); Grade 2 (moderate) n=193 (14.1%); Grade 3 (severe) n=82 (6.0%) | Median (IQR) 5.5 (10.8) years | NR. Deaths for the entire follow-up period n=1099 (80.2%) |
|  | **Hoban 2017** | EV:Michigan | LSCC | 60.0 (10.2) | 190 (77.2%) | TNM-version NR: I n=60 (24.3%); II n=34 (13.8%); III n=53 (21.5%); IV n=99 (40.2%) | T1 n=64 (25.9%); T2 n=52 (21.1%); T3 n=71 (28.8%); T4 n=59 (23.9%). N0 n=156 (63.4%); N1 n=26 (10.5%); N1b n=1 (0.4%); N2 n=1 (0.4%); N2a n=2 (0.8%); N2b n=24 (9.7%); N2c n=33 (13.4%); N3 n=3 (1.2%) | Current n=166 (67.4%); Former n=61 (24.7%); Never n=18 (7.3%) | NR | IC n=70 (28.4%); Surgery n=65 (26.4%); CRT n=56 (22.7%); RT only n=55 (22.3%) | ACE-27: Grade 0 (none) n=49 (19.9%); Grade 1 (mild) n=112 (45.5%); Grade 2 (moderate) n=60 (24.3%); Grade 3 (severe) n=17 (6.9%) | Median 60 months | 5-year OS (95% CI) = 71.9% (65-78), n=69 deaths |
| **Egelmeer** | **Egelmeer 2011** | Dev: MAASTRO^ | LSCC | Median 65.0 years, (range 31–91) | 883 (88.8%) | NR | T1 n=528 (53.1%); T2 n=264 (26.6%); T3 n=131 (13.2%); T4 n=71 (7.1%). N0 n=894 (89.9%); N1 n=45 (4.5%); N2 n=42 (4.2%); N3 n=11 (1.1%); Missing n=2 (0.2%) | NR | NR | RT only (100%) | NR | Median 72 months (range 2– 72) | 2-year OS = 82.8%, n=171 deaths. 5-year OS=67.7%, n=321 death. 2-years LC= 71.0%, n=179. 5-years LC= 54.0%, n=220 |
|  |  | E1: Leuven | Glottic and Supraglottic SCC | 18–60 years: n=40 (36.7%), > 60 years: n=69 (63.3%) | 99 (90.8%) | NR | T1 n=45 (41.3%); T2 n=30 (27.5%); T3 n=24 (22.0%); T4 n=10 (9.2%). N0 n=82 (75.2%); N1 n=6 (5.5%); N2 n=18 (16.5%); N3 n=3 (2.8%) | NR | NR | RT only (100%) | NR | NR | NR |
|  |  | E2: VU | LSCC | NR | 154 (86.5%) | NR | T1 n=67 (37.6%); T2 n=91 (51.1%); T3 n=16 (9.0%); T4 n=4 (2.2%). N0 n=165 (92.7%); N1 n=5 (2.8%); N2 n=6 (3.4%); N3 n=0; Missing n=2 (1.1%) | NR | NR | RT only (100%) | NR | NR | NR |
|  |  | E3: NKI/AVL | Glottic and Supraglottic SCC | 18–60 years: n=75 (36.5%), >60 years: n=130 (63.5%) | 162 (79.1%) | NR | T1 n=86 (41.9%); T2 n=119 (58.1%);T3 n=0; T4 n=0. N0 n=184 (89.8%); N1 n=6 (2.9%); N2 n=11 (5.4%); N3 n=4 (1.9%) | NR | NR | RT only (100%) | NR | NR | NR |
|  |  | E4: Manchester (1998-2005) | Glottic SCC | 18–60 years: n=154 (38.2%); >60 years: n=249 (61.8%) | 357 (88.6%) | NR | T1 n=252 (62.5%); T2 n=124 (30.8%); T3 n=27(6.7%); T4 n=0. N0 n=398 (98.8%); N1 n=1 (0.2%); N2 n=3 (0.7%); N3 n=1 (0.2%) | NR | NR | RT only (100%) | NR | NR | NR |
|  | **Hoban 2017** | E5: Michigan | LSCC | 60.0 (10.2) | 190 (77.2%) | TNM-version NR: I n=60 (24.3%); II n=34 (13.8%); III n=53 (21.5%); IV n=99 (40.2%) | T1 n=64 (25.9%); T2 n=52 (21.1%); T3 n=71 (28.8%); T4 n=59 (23.9%). N0 n=156 (63.4%); N1 n=26 (10.5%); N1b n=1 (0.4%); N2 n=1 (0.4%); N2a n=2 (0.8%); N2b n=24 (9.7%); N2c n=33 (13.4%); N3 n=3 (1.2%) | Current n=166 (67.4%); Former n=61 (24.7%); Never n=18 (7.3%) | NR | IC n=70 (28.4%); Surgery n=65 (26.4%); CRT n=56 (22.7%); RT only n=55 (22.3%) | ACE-27: Grade 0 (none) n=49 (19.9%); Grade 1 (mild) n=112 (45.5%); Grade 2 (moderate) n=60 (24.3%); Grade 3 (severe) n=17 (6.9%) | Median 60 months | 5-year OS (95% CI) = 71.9% (65-78), n=69 deaths |
|  | **Aly 2021^+^** | E6: NSW | LSCC | Median 69.0 years, (range 46.8–91.2) | 91 (86.7%) | NR | T1 n=23 (21.9%); T2 n=41 (39%); T3 n=33 (31.4%); T4 n=8 (7.6%). N0 n=75 (71.5%); N1 n=8 (7.6%); N2 n=21 (20%); N3 n=1 (1%) | NR | NR | RT only n=69 (65.7%), CRT n=36 (34.3%) | NR | Median 46 months (range 7-121) | 2-year OS = 75%. 2-year LR=68.2%, n=15 |
|  | **Ron Hansen 2019^+^** | E7: DAHNCA (2005-2015) | LSCC | NR | NR | NR | NR | NR | NR | NR | NR | Median 2.6 years | NR |
|  | **Hansen 2022** | E8: Odnese/DAHNCA (2005-2018)^^^ | LSCC | Median 67 years (range 37–96) | 547 (81%) | NR | T1 n=258 (38%); T2 n=242 (36%); T3 n=114 (17%); T4 n=58 (9%). N0 n=493 (73%); N+ n=179 (27%) | NR | NR | RT (100%) | NR | NR (< 60 months) | NR |
|  |  | E9: Manchester (2005-2018) | LSCC | Median 67 years (range 29–96) | 339 (80%) | NR | T1 n=133 (31%); T2 n=166 (39%); T3 n=86 (20%); T4 n=25 (6%); Missing n=13 (3%). N0 n=348 (82%); N+ n=70 (17%); Missing n=5 (1%). | NR | NR | RT (100%) | NR | NR (< 60 months) | NR |
| **Emerick** | **Emerick 2013^%^** | Dev: SEER | SCC originating from oropharynx, nasopharynx, larynx, oral cavity, and hypopharynx | NR | NR | NR | NR | NR | NR | NR | Not reported | NR | NR |
|  | **Hoban 2017** | EV: Michigan | LSCC | 60.0 (10.2) | 190 (77.2%) | TNM-version NR: I n=60 (24.3%); II n=34 (13.8%); III n=53 (21.5%); IV n=99 (40.2%) | T1 n=64 (25.9%); T2 n=52 (21.1%); T3 n=71 (28.8%); T4 n=59 (23.9%). N0 n=156 (63.4%); N1 n=26 (10.5%); N1b n=1 (0.4%); N2 n=1 (0.4%); N2a n=2 (0.8%); N2b n=24 (9.7%); N2c n=33 (13.4%); N3 n=3 (1.2%) | Current n=166 (67.4%); Former n=61 (24.7%); Never n=18 (7.3%) | NR | IC n=70 (28.4%); Surgery n=65 (26.4%); CRT n=56 (22.7%); RT only n=55 (22.3%) | ACE-27: Grade 0 (none) n=49 (19.9%); Grade 1 (mild) n=112 (45.5%); Grade 2 (moderate) n=60 (24.3%); Grade 3 (severe) n=17 (6.9%) | Median 60 months | 5-year OS (95% CI) = 71.9% (65-78), n=69 deaths |
| **Lustberg** | **Lustberg 2016** | Dev: MAASTRO^ | LSCC | >60 years: n=621 (63%) | 870 (89%) | NR | T1 n=524 (54%); T2 n=260 (27%); T3 n=128 (13%); T4 n=66 (7%). N0 n=884 (90%); N+ n=98 (10%) | NR | NR | RT only (100%) | NR | NR | NR |
|  |  | E1: Wollongong | LSCC | >60 years: n=37 (71%) | 47 (90%) | NR | T1 n=18 (35%); T2 n=11 (21%); T3 n=14 (27%); T4 n=7 (13%); missing n=2 (4%). N0 n=41 (79%); N+ n=11 (21%) | NR | NR | RT only (100%) | NR | NR | NR |
|  |  | E2: RTOG 91-11 | LSCC | >60 years: n=81 (46%) | 136 (77%) | NR | T1 n=0; T2 n=18 (10%); T3 n=144 (81%); T4 n=15 (8%). N0 n=92 (52%); N+ n=85 (48%). | NR | NR | RT only (100%) | KPS >60 (100%) | NR | NR |
| **Petersen** | **Petersen 2018** | Dev: TNCR | Advanced LSCC (T3/T4, N0/N+, M0) | Mean 64, range (28–100) | 2705 (78.6%) | NR | T3N0 n=1237 (35.9%); T3N1 n=681 (19.8%); T4N0 n=887 (25.8%); T4N1 n=637 (18.5%). All M0 | NR | NR | Surgery (TL) n=1168 (33.9%); RT n=2009 (58.4%); CRT n=265 (7.7%) | NR | NR | NR |
|  |  | EV: Five pooled cohorts | Advanced LSCC (T3/T4, N0/N+, M0) | Mean 62, range (16–92) | 2705 (78.6%) | NR | T3N0 n=282 (36.6%); T3N1 n=145 (18.8%); T4N0 n=174(22.6%); T4N1 n=169 (21.9%). All M0 | NR | NR | Surgery (TL) n=311 (40.4%); RT n=281 (36.5%); CRT n=213 (27.7%) | NR | NR | NR |
| **Tian** | **Tian 2021** | Dev: SEER | HPSCC | ≤50 y: n=171 (9.7%), 51–60 y: n=562 (32%), 61–70 y: n=607 (34.5%), >70 y: n=418 (23.8%) | 1466 (83.4%) | TNM-7: I n=56 (3.2%); II n=160 (9.1%); III n=321 (18.3%); IV-A n=865 (49.2%); IV-B n=213 (12.1%); IV-C n=143 (8.1%) | T1 n=173 (9.8%); T2 n=626 (35.6%); T3 n=438 (24.9%); T4a n=350 (19.9%); T4b n=171 (9.7%). N0 n=428 (24.3%); N1 n=333 (18.9%); N2 n=894 (50.9%); N3 n=103 (5.9%). M0 n=1615 (91.9%); M1 n=143 (8.1%) | NR | NR | Surgery n=64 (3.6%); RT n=1440 (81.9%); Surgery + RT n=237 (13.5%); Both not given n=17 (1%). CT n=1244 (70.8%) | NR | NR | NR |
|  |  | IV: SEER | HPSCC | ≤50 y: n=35 (8%), 51–60 y: n=35 (8%), 61–70 y: n=142 (32.3%), >70 y: n=106 (24.1%) | 359 (81.6%) | TNM-7: I n=25 (5.7%); II n=44 (10%); III n=75 (17%); IV-A n=210 (47.7%); IV-B n=47 (10.7%); IV-C 39 (8.9%) | T1 n=44 (10%); T2 n=152 (34.5%); T3 n=114 (25.9%); T4a n=93 (21.1%); T4b n=37 (8.4%). N0 n=118 (26.8%); N1 n=68 (15.5%); N2 n=230 (52.3%); N3 n=24 (5.5%). M0 n=401 (91.1%); M1 n=39 (8.9%) | NR | NR | Surgery 27 (6.1%); RT n=337 (76.6%); Surgery + RT n=72 (16.4%); Both not given n=4 (0.9%). CT n=300 (68.2%) | NR | NR | NR |
|  |  | EV: Fudan University | HPSCC | ≤50 y: n=29 (12.4%), 51–60 y: n=86 (36.9%), 61–70 y: n=100 (42.9%), >70 y: n=18 (7.7%) | 229 (98.3%) | TNM-7: I n=0 (0%); II n=0 (0%); III n=52 (22.3%); IV-A n=164 (70.4%); IV-B n=17 (7.3%); IV-C n=0 (0%) | T1 n=6 (2.6%); T2 n=65 (27.9%); T3 n=82 (35.2%); T4a n=80 (34.3%); T4b n=0 (0%). N0 n=24 (10.3%); N1 n=69 (29.6%); N2 n=123 (52.8%); N3 n=17 (7.3%). All patients M0 | NR | NR | Surgery n=0 (0%); RT n=51 (21.9%); Surgery + RT n=182 (78.1%); Both not given n=0 (0%). CT n=193 (82.8%) | NR | Median 27.9 months (IQR 19.3-38.3) | 3-year OS (95% CI) = 64.6% (56.6-72.6) |
| **Zhu** | **Zhu 2020** | Dev: SEER | Advanced LSCC (T3/T4, N0/N+): n=5899 (97.2%), Other: n=171 (2.8%) | NR | 4854 (80%) | NR | T3 n=3483 (57.4%); T4 n=2587 (42.6%). N0 n=3104 (51.1%); N1+ n=2966 (48.9%) | NR | NR | Surgery n=2806 (46.2%); RT n=5176 (85.3%); CT n=3843 (63.3%) | NR | Median 29 months | 5-year OS (42.9%) |
|  |  | EV: Fudan University | Advanced LSCC (T3/T4, N0/N+): n=605 (97.3%), Other: n=17 (2.7%) | NR | 604 (97.1%) | NR | T3 n=473 (76.0%); T4 n=149 (24.0%). N0 n=460 (74.0%); N1+ n=162 (26.0%) | NR | NR | Surgery n=622 (100.0%); RT n=129 (20.7%) | NR | Median 50 months | 5-year OS (44.6%) |
| **RSM** | | | | | | | | | | | | | |
| **Ho** | **Ho 2018** | Dev: NCDB | LSCC: n=7019 (84.05%), HPSCC: n=1332 (15.95%) | 61.0 (10.1) | 6499 (77.8%) | TNM-8: stage NR | T1 n=515 (6.2%); T2 n=1330 (15.9%); T3 n=2549 (30.5%); T4 n=3957 (47.4%). pN0 n= 3641 (43.6%); pN2c n=1508 (18.1%). Rest of N classification NR. M0 (100%) | NR | NR | Surgery 100%; Surgery + PORT n=4886 (58.5%); Surgery + POCT n=2263 (27.1%) | CCI: Grade 0 n=5466 (65.45%); Grade 1 n=2187 (26.19%); Grade >= 2 n=698 (8.36%) | NR | NR |
|  | **Choi 2019** | EV: Seoul | LSCC: n=98 (69.5%), HPSCC: n=43 (30.5%) | Median 65.0 years, (range 33–78) | 132 (93.6%) | TNM-8: stage NR | T-classification NR. pN0 n=66 (46.8%); pN1 n=21 (14.9%); pN2a n=8 (5.7%); pN2b n=20 (14.2%); pN2c n=7 (5.0); pN3a n=0; pN3b n=19 (13.5%). All patients M0 | ≥ 20 PYI n=107 (75.9%) | n=105 (74.5%) | Surgery only n=60 (42.6%); Surgery + PORT only n=67 (47.5%); Surgery + POCRT n=14 (9.9%) | CCI: ≥ grade 1 n=60 (42.6%). ECOG performance scale ≥ grade 1 n=23 (16.3%) | Median 60 months, (range 24–134) | All-cause mortality n=59 (41.8%) |
| **Lacy** | **Lacy 1998** | Dev: Missouri | LSCC | 62 (10) | 102 (82%) | TNM-4: I n=35 (28.2%); II n=28 (22.6%); III n=31 (25%); IV n=30 (24.2%) | NR | n=111 (89.5%) | NR | Surgery only n=25 (20.2%); RT only n=49 (39.5%); Surgery + RT n=46 (37.1%); other n=3 (2.4%) | Modified KFI stage 3 (severe): n=11 (8.9%) | NR. All >2 years | 2-year OM after recurrence n=74 (59.7%) |
|  | **Lacy 2004** | EV: Melbourne | LSCC | 62 (9) | 60 (90%) | TNM-5: I n=18 (26.9%); II n=13 (19.4%); III n=21 (31.4%); IV n=15 (22.4%) | NR | NR | NR | Surgery alone n=41 (61.2%); RT only n=19 (28.4%); Surgery + RT n=7 (10.5%) | NR | NR. All >2 years, n=66 (98%) >5-year follow-up | 2-year OM after recurrence n=33 deaths (49.3%) |

Dev, Development cohort; IV, internal validation; EV, external validation; IPM, Individualised prediction models; RSM, Risk stratification models; HPSCC, hypopharyngeal squamous cell carcinoma; LSCC, laryngeal squamous cell carcinoma; n, numbers; SD, standard deviation; CI, confidence interval; T,N,M Tumor, Node and Metastasis classification, TNCR, The Netherlands cancer registry; NCDB, National cancer database (USA); SEER, Surveillance, Epidemiology, and End Results database (USA); DAHANCA, The Danish Head and Neck cancer Study Group database; NR, Not Recorded; IC, induction chemotherapy; CT, chemotherapy; RT, radiotherapy; PORT, post-operative RT; CRT, chemoradiotherapy; POCRT, post-operative CRT; TL, total laryngectomy; ACE-27, Adult Co-Morbidity Evaluation; KPS, The Karnofsky Performance Scale Index; KFI, Kaplan-Feinstein Comorbidity Index; CCI, Charlson Comorbidity Index; OS, overall survival; LC, local control; LR, local recurrence; OM, overall mortality.

^%^ Models developed using mixed head and neck cancer cohorts (not only LSCC/HPSCC)

^ Potentially overlapping cohorts

^+^ Models reported in conference abstracts
